# snoRNAs are a novel class of biologically relevant Myc targets

**DOI:** 10.1186/s12915-015-0132-6

**Published:** 2015-04-16

**Authors:** Eva K Herter, Maria Stauch, Maria Gallant, Elmar Wolf, Thomas Raabe, Peter Gallant

**Affiliations:** Theodor Boveri Institute, Biocenter, University of Würzburg, Würzburg, Germany; Comprehensive Cancer Center Mainfranken, Würzburg, Germany; Institute for Medical Radiation and Cell Research, Würzburg, Germany

**Keywords:** Myc, Transcription, snoRNA, Ribosome, Growth, *Drosophila*

## Abstract

**Background:**

Myc proteins are essential regulators of animal growth during normal development, and their deregulation is one of the main driving factors of human malignancies. They function as transcription factors that (in vertebrates) control many growth- and proliferation-associated genes, and in some contexts contribute to global gene regulation.

**Results:**

We combine chromatin immunoprecipitation-sequencing (ChIPseq) and RNAseq approaches in *Drosophila* tissue culture cells to identify a core set of less than 500 Myc target genes, whose salient function resides in the control of ribosome biogenesis. Among these genes we find the non-coding snoRNA genes as a large novel class of Myc targets. All assayed snoRNAs are affected by Myc, and many of them are subject to direct transcriptional activation by Myc, both in *Drosophila* and in vertebrates. The loss of snoRNAs impairs growth during normal development, whereas their overexpression increases tumor mass in a model for neuronal tumors.

**Conclusions:**

This work shows that Myc acts as a master regulator of snoRNP biogenesis. In addition, in combination with recent observations of snoRNA involvement in human cancer, it raises the possibility that Myc’s transforming effects are partially mediated by this class of non-coding transcripts.

**Electronic supplementary material:**

The online version of this article (doi:10.1186/s12915-015-0132-6) contains supplementary material, which is available to authorized users.

## Background

Growth and proliferation are fundamental for all life. In metazoans, these processes need to be coordinated with animal development and homeostasis [1]. One important factor in the control and coordination of growth is the Myc family of oncogenes (represented by a single protein in insects, simply called Myc, and three major paralogs in vertebrates: c-, N- and L-Myc). Myc’s activity is essential for normal animal development in insects and in vertebrates (reviewed in [2,3]). A moderate reduction of Myc levels in *Drosophila* results in small cells with small nucleoli, reduced organismal growth and adult size, delayed overall development and female sterility [4,5]. Fruit flies lacking all Myc activity fail to undergo normal growth and mostly die during early larval stages [6,7]. Conversely, Myc overexpression in vertebrates (and to some extent in *Drosophila*) leads to unscheduled cellular growth and proliferation [8]. Consistent with this, mutational activation of Myc, or of upstream signaling pathways, is frequently found in human cancers and causally contributes to this disease [9]. Knowledge of the relevant Myc targets, therefore, is essential to understand the processes of Myc-dependent growth in normal and pathological situations.

While Myc can control a large number of genes in vertebrates [10-13], the number of Myc targets is still open in the simpler model *Drosophila*. Several studies have characterized Myc-regulated genes [14-16], but they have not comprehensively addressed which of these genes are controlled by direct Myc binding, nor have they included genes that do not code for proteins. However, recent studies have revealed the abundance and variety of non-coding transcripts in the eukaryotic genome, and have emphasized the biological importance of various such transcripts [17,18]. Some non-coding RNAs have already been identified as physiologically relevant Myc targets in vertebrates (rRNAs, tRNAs, miRNAs; see [19]), raising the possibility that the biological activities of Myc might be mediated (in part) by these and additional non-protein coding transcripts.

We, therefore, set out to extend the search for Myc targets to non-polyadenylated transcripts, using a combination of RNAseq and chromatin immunoprecipitation sequencing (ChIPseq) approaches in cultured *Drosophila* cells. This approach led to the identification of a core set of less than 500 Myc targets. The majority of these targets control ribosome biogenesis and translation, in good agreement with earlier reports [8,20,21]. In addition to the previously identified mRNAs, however, we identified small nucleolar RNAs (snoRNAs) as a novel class of Myc targets. The *Drosophila* genome encodes 288 snoRNAs (Flybase release FB2014_6; [22]), most of which fall into one of two classes: the 60 to 100 nucleotides long box C/D snoRNAs and the 130 to 160 nucleotides long box H/ACA snoRNAs. Upon association with a small set of specific proteins, these two types of snoRNAs form small nucleolar riboproteins (snoRNPs) that catalyze 2’-O-methylation and pseudouridylation, respectively. Their best characterized targets are small nuclear RNAs (snRNAs) and ribosomal RNAs, where many of these post-transcriptional modifications cluster in functionally important regions and contribute to efficient ribosome biogenesis and/or function. In addition, snoRNAs have been shown to affect other biological processes, such as RNA editing, alternative splicing, and gene silencing (reviewed by [23,24]). Intriguingly, the snoRNA associated proteins all are encoded by core Myc targets, as are several of the factors involved in snoRNP processing [8]. These findings indicate that Myc acts as a master regulator of snoRNP biogenesis, and they suggest a biological mechanism that ensures the stoichiometry of RNA and protein components of snoRNPs. At the same time, they reinforce the notion that snoRNP generation, and hence ribosome biogenesis, constitutes the core function of *Drosophila* Myc. We further provide evidence that vertebrate Myc also controls snoRNA expression. Finally, we show that the snoRNA host gene Uhg1 is important for normal animal growth, and that overexpression of different Uhg genes enhances tumor growth.

## Results

### Myc directly binds a core set of sites in *Drosophila*

**Figure 1:**
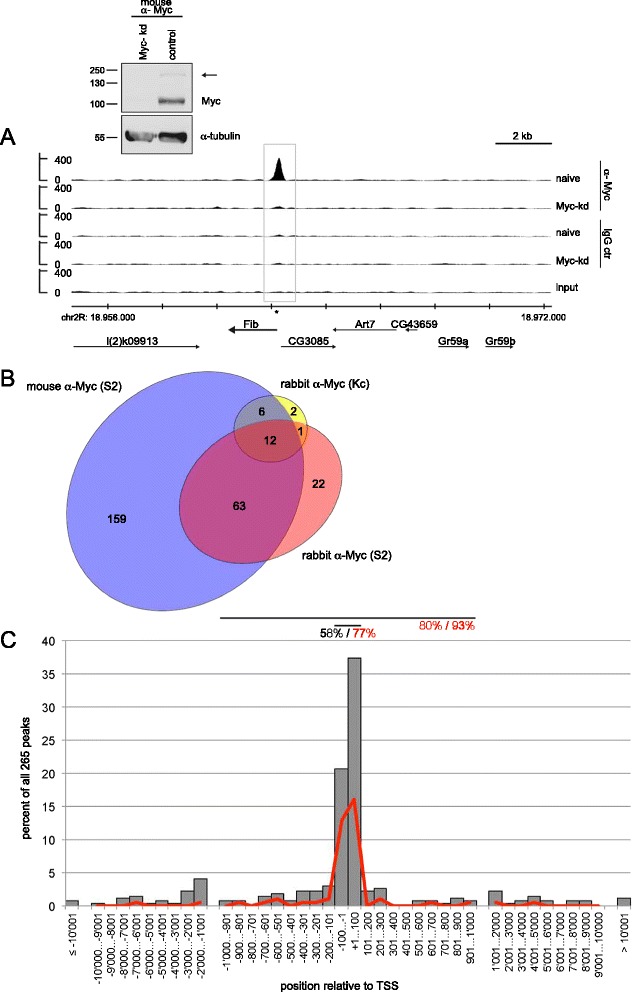
Myc binding sites in *Drosophila*. **A)** Myc binding to the Fibrillarin locus as an example of a binding site in S2 cells. Strong binding is observed with mouse α-Myc antibodies in naïve S2 cells (1^st^ lane), but not upon Myc depletion (2^nd^ lane) or with control mouse IgG (3^rd^ and 4^th^ lane); the grey box marks the Myc binding peak as called by the software MACS, the asterisk shows a consensus E-box (‘CACGTG’). Chromosomal coordinates (on chromosome 2R) are indicated below the traces, as are the extents and orientations of genes in this region. The Western blot illustrates the efficiency of Myc depletion; the arrowhead points to a band presumably arising from post-translational modification of Myc. Molecular weight markers (in kD) are indicated. **B)** Overlaps of Myc binding sites from three different ChIPseq experiments, using either mouse monoclonal or rabbit polyclonal anti-Myc antibodies, and chromatin from S2 or Kc167 cells. Only peaks called by the software as ‘significant’ were considered, that is, with false discovery rate (FDR) of <10%. **C)** Position of Myc binding peaks relative to the nearest transcription start site (TSS). Data are distributed in 100-nt bins for a distance of up to 1,000 nt from the TSS and in 1,000-nt bins for distances between 1,000 and 10,000 nt, and shown as percent of all 265 peaks. Grey bars show all Myc binding peaks, the red line only the Myc binding peaks containing a canonical E-box within a 100 nt window straddling the Myc binding summit. Horizontal lines above the graph illustrate windows of +/− 100 nt and +/− 1,000 nt around the TSS, containing 58% and 80% of all Myc peaks, and 77% and 93% of the Myc peaks with canonical E-boxes, respectively.

In order to establish a core set of genes directly controlled by Myc in *Drosophila*, we performed ChIPseq experiments in S2 cells using a mouse monoclonal anti *Drosophila* Myc antibody [25]. To control for background signal, we used non-immune mouse immunoglobulin G (IgG) in parallel experiments, and we repeated both anti-Myc and control IgG ChIPs with cells that had been depleted of Myc. This set of experiments resulted in the identification of 240 peaks that are specifically bound by Myc in naïve S2 cells but not in Myc-depleted cells, and that are not recognized by control IgGs (Figure 1A, Additional file 1: Table S1). Since these ChIPs relied on a monoclonal antibody, it is conceivable that some Myc binding sites were missed due to epitope masking. To exclude this possibility, we conducted another set of ChIPseq experiments with a rabbit polyclonal anti *Drosophila* Myc antibody [26] and chromatin from S2 cells, again using species-matched non-immune IgGs as control. This approach yielded 98 specifically Myc-bound peaks, most of which (75) overlapped with the Myc-binding sites identified above (Figure 1B, Additional file 1: Table S1). Given this coincidence between the two antibodies, we are confident that we have identified the majority of Myc bound genes, and (based on the negative controls) that these genes represent *bona fide* Myc-binding sites.

This number of Myc-binding sites is consistent with what was found in an earlier study that used DamID for assessing Myc binding [14], but it is considerably lower than the nearly 4,000 sites that were recently reported for a ChIPseq study of *Drosophila* Kc167 cells [27]. Several possibilities can be considered for this discrepancy. First, although both Kc167 and S2 cells are hematopoietic cells of embryonic origin, it is conceivable that they differ in their molecular characteristics. To address this possibility, we carried out ChIPseq experiments with rabbit anti-Myc antibodies and control IgGs using chromatin from Kc167 cells. This led to the identification of 279 Myc-binding sites (110 of which overlap peaks that were found in S2 cells). However, in these experiments we observed considerably higher background binding than in S2 cells, and, therefore, only 21 binding sites were statistically significant (false discovery rate (FDR) <10%), 19 of which overlap peaks in S2 cells. This similarity with the results in S2 cells argues against major differences between the cell lines (Figure 1B, Additional file 1: Table S1). Second, Yang *et al*. used a different antibody (Santa Cruz rabbit anti *Drosophila* Myc) which might have higher affinity and/or recognize other epitopes on Myc, thus allowing the identification of additional *bona fide* Myc-binding sites. Alternatively, since there are no published data showing the specificity of this antibody, or of control ChIPs with non-immune IgG, or control ChIPs from Myc-depleted cells, some of the 4,000 peaks might also reflect non-specific binding of the antiserum to chromatin. To address such a possibility, we prepared chromatin from naïve and from Myc-depleted S2 cells, and conducted ChIP experiments with this commercial antiserum. As expected, the ChIP signals at the high-affinity Myc targets nop5 and Uhg1 (see below) are strongly reduced upon Myc depletion (Additional file 2: Figure S1B). In contrast, very poor or no reduction is observed at 18 sequences that were identified as Myc binding sites by Yang *et al*. but not in our experiments (Additional file 2: Figure S1A, B). This non Myc-specific ChIP signal presumably corresponds to a background reactivity of the commercial antibody, which is unaffected by the Myc knock-down even though Myc protein is depleted highly efficiently (arrow in Additional file 2: Figure S1B). By inference, we consider it most likely that most of the sequences that were not identified in our experiments do not specifically bind to Myc in *Drosophila*. In our following analysis we, therefore, focus on the 265 sites we have identified in our combined ChIPseq experiments as the core set of Myc binding sites in *Drosophila* (all sites that were specifically bound in at least one experiment in S2 or Kc167 cells, with an FDR <10%; Additional file 1: Table S1).

### Ribosome biogenesis and ribosome protein genes are the core Myc targets

In a first step, we determined the position of the binding sites relative to annotated genes. Fifty-eight percent of all sites map within 100 bp from the nearest transcription start site (TSS; grey bars in Figure 1C), and 80% of the sites are less than 1,000 bp from a TSS, suggesting that Myc preferentially binds to promoter-proximal sequences. This preference is even more striking when only Myc peaks are considered that cover a canonical E-box, with 77% of these peaks localizing to within 100 bp of a TSS (red line in Figure 1C). Due to the high gene density in *Drosophila*, several Myc binding sites are located close to more than one TSS, so that altogether 279 genes are potentially affected by Myc binding (see below, Additional file 1: Table S2).

To determine which of these (or of any other) genes indeed require Myc for their correct expression we conducted RNAseq experiments in S2 cells. For this purpose, we transfected dsRNA directed against Myc (or GFP as a control) into *Drosophila* S2 cells and harvested the cells 24 hours later for total RNA isolation, by which time Myc protein levels were strongly reduced (Additional file 2: Figure S2). In order to assess both polyadenylated and non-adenylated transcripts, we then depleted the samples of ribosomal RNA and processed the remainder for deep sequencing. The bioinformatic analysis of biologically independent triplicates (both for Myc knock-down and control) resulted in 8,019 genes with detectable expression in S2 cells and a minimal transcript length of 125 nt. Of these, 281 genes were differentially expressed upon Myc depletion (see Methods; Additional file 1: Table S3). The majority of these genes, 240, is downregulated upon Myc depletion. Among these, the poly-adenylated transcripts show a good overlap with previously established lists of Myc-activated genes that were obtained in different settings (Additional file 2: Figure S3A; [15,16,28]). Note that, in contrast to the Myc-activated genes, Myc-repressed genes show only poor overlap between different data sets (Additional file 2: Figure S3B). Taken together, these observations validate the current RNAseq data set.

A comparison of the binding and expression data shows that 139 of the Myc-bound genes are downregulated by at least one third upon Myc-knockdown and 59 are upregulated by at least one third (Additional file 2: Figure S4A, B). The remaining transcripts were either not detected in our RNAseq experiments (due to low expression levels in S2 cells or transcript sizes below the 125 nt cutoff) or they showed lower responses to altered Myc levels (some of the corresponding transcripts might be too stable to show a significant decline over the course of the experiment). The following analysis focuses on the 139 genes that are bound by Myc and require Myc for their full expression and that we, therefore, consider to be the core set of directly Myc-activated genes. The overwhelming majority of these genes (114, 82%) functions in ribosome biogenesis and in translation (Table 1, Additional file 1: Table S4). Myc controls several steps in this pathway, starting with the synthesis of ribosomal RNA by RNA polymerase I (see [8]). Consistent with earlier reports [28], we did not observe direct binding of Myc to rRNA loci in our ChIPseq experiments, but Myc increases the activity of RNA polymerase I by inducing some of its components or cofactors (for example, RpI135; Additional file 1: Table S4). Numerous Myc targets then control the post-transcriptional modifications of rRNA in general (15 genes coding for components of small nucleolar ribonucleoproteins, RNPs), and the maturation of the 40S subunit (18 genes) or 60S subunit (17 genes). Myc further induces the expression of ribosomal protein genes (19, 21 and 2 genes coding for components of the small and large ribosomal subunits and the mitochondrial ribosome, respectively), of translation factors (6 genes) and of genes involved in tRNA maturation (6 genes). Myc has also been reported to promote RNA polymerase III activity via an interaction with its co-factor Brf and, therefore, increases the amounts of tRNAs and other Pol III products [29-31]; because of their small size these transcripts were not contained in our analysis. We also did not observe any significant enrichment of Myc at the 306 annotated tRNA loci in our ChIPseq data (data not shown). Presumably, Myc does not contact DNA directly at these sites; indeed, Myc does not require its DNA binding domain nor its classical dimerization partner Max to activate RNA polymerase III [30], and most tRNA loci (295 of 306) also do not contain any consensus Myc binding site (E-box; data not shown).

**Table 1:** **Categories of directly Myc-activated genes**

**Function**	**Genes**
RNA Pol I activity	7
snoRNP function	15
40S subunit assembly, processing, maturation	18
60S subunit assembly, processing, maturation	17
processing of both subunits	3
40S ribosomal subunit	19
60S ribosomal subunit	21
Mitochondrial ribosome	2
Translation factors	6
tRNA processing, maturation	6
***Total RiBi and translation***	***114***
Metabolism	5
Transcription, RNA processing	5
Mitochondrial function	4
Other, unknown	11

RiBi, ribosome biogenesis; snoRNP, small nucleolar ribonucleoproteins.

The observation that the majority of Myc-activated genes controls ribosome biogenesis and translation indicates that these are the main and presumably primordial functions of Myc proteins. At the same time, they emphasize Myc’s central role for the production (and to some extent, activity) of ribosomes. Indeed, of 286 genes that were recently identified as essential for rRNA processing in human cells [32], the homologs of 43 count among these core Myc targets in *Drosophila*. The situation is particularly striking for snoRNPs, whose best-characterized activities reside in the post-transcriptional modifications of rRNAs. All protein components of snoRNPs are known to be Myc targets, and our analysis now uncovers a large and novel class of Myc targets: the non-coding snoRNAs.

### A novel class of Myc targets: snoRNA and Uhg genes

**Figure 2:**
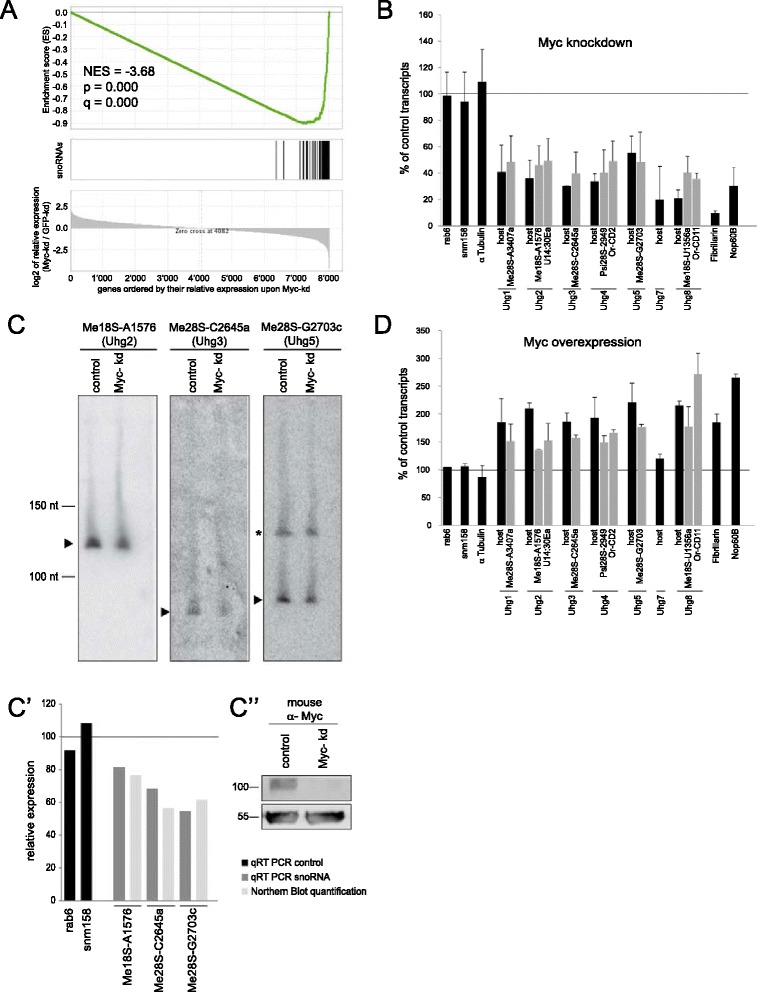
Effect of altered Myc levels on Uhg and snoRNA expression. **A)** Gene set enrichment analysis (GSEA) of snoRNAs. The 8,019 detectable genes are ordered by relative expression (log2 of the ratio of Myc-knockdown over GFP-knockdown) from left to right, and all detectable snoRNAs are mapped onto these genes (black vertical bars). NES indicates the normalized enrichment score, p the nominal p-value and q the false discovery rate. **B and C)** RNA levels were assayed 24 hours after addition of Myc-dsRNA to S2 cells by quantitative PCR **(B)** and Northern blotting **(C)**; of ten investigated snoRNAs, five gave no signal or such a weak signal that a reduction in response to Myc-knockdown could not be reliably quantified, four were clearly reduced upon Myc-knockdown and one was unaffected (see Additional file 1: Table S5). **D)** qPCR assays carried out six hours after addition of 125 μM CuSO_4_ to S2-Myc cells to induce Myc overexpression. Error bars show standard deviations of biologically independent duplicates. Selected snoRNAs are grouped with the transcripts of their host Uhg genes. Panel **C**, arrowheads point to mature snoRNAs; the identity of the cross-reactive slower migrating band in the Me28S-G2703c blot (asterisk) is unclear. The locations of molecular weight DNA markers are indicated. The same RNA samples were analyzed by reverse transcription and quantitative PCR for the reference genes *snm158* and *rab6*, as well as for the snoRNAs. **C’,** qPCR results and quantification of the Northern blot bands. **C”,** part of each sample that was not used for RNA isolation (shown in panels C and C’) was analyzed by Western blotting with mouse anti-Myc antibody (top) and mouse anti-α-Tub84B (bottom). The experiment shown in panels **C**, C’ and C” is representative of biologically independent duplicates. snoRNA, small nucleolar RNA; Uhg, U-snoRNA host genes.

After Myc depletion 240 genes are significantly downregulated (*P* <0.05) – and 36 of these do not code for proteins, but instead for snoRNAs. The *Drosophila* genome is predicted to encode 288 snoRNAs ([33] and FlyBase), ranging in size from 46 to 316 nt. Strikingly, all of the 103 snoRNAs that pass our 125 nt size cutoff and are detectably expressed in S2 cells are strongly downregulated upon Myc depletion (Figure 2A and Additional file 1: Table S3). This includes most of the box H/ACA snoRNAs, but the majority of the box C/D snoRNAs are smaller than 125 nt and, therefore, not detectable in these RNAseq experiments. However, qPCR and Northern blot analysis of several small box C/D snoRNAs confirmed that they, too, are significantly depleted upon Myc knockdown (see below), suggesting that Myc affects snoRNA levels in general. This dramatic impact of Myc on snoRNA levels can be explained by a combination of different mechanisms. First, most *Drosophila* snoRNA genes are encoded in introns of other genes, from which the mature snoRNAs are excised [34]. Many of the 84 different host genes are *bona fide* Myc targets (Additional file 2: Figure S5), and Myc’s impact on host gene expression is likely to be reflected in reduced levels of the intron-encoded snoRNAs. Second, snoRNAs function as part of snoRNPs, whose known protein components all are Myc targets (see Additional file 1: Table S4 and e.g. [23]). Hence, Myc downregulation is expected to deplete snoRNPs and, as a consequence, destabilize the corresponding snoRNAs [35]. Third, Myc also controls the expression of genes that are dedicated to the synthesis of snoRNAs, the so-called Uhg genes (‘U-snoRNA host genes’; [33,36]). The following analysis focuses on Myc’s transcriptional effects on these Uhg genes.

**Figure 3:**
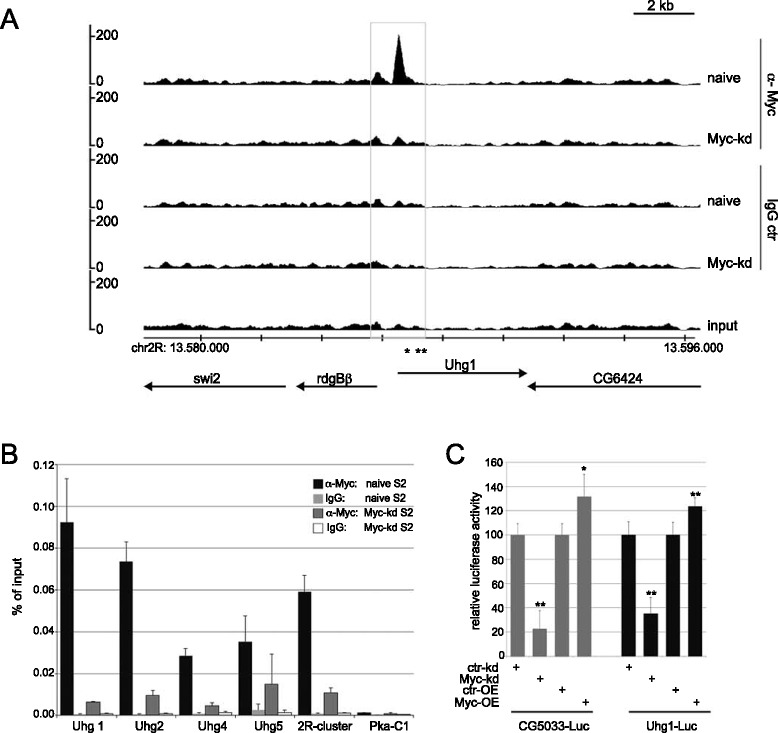
Myc directly binds to and controls Uhg promoters. **A)** ChIPseq profile for the Uhg1 locus in S2 cells. Labeling conventions are as in Figure 1A. **B)** ChIP-qPCR of Uhg loci. Chromatin was isolated from naïve S2 cells or from S2 cells depleted of Myc, precipitated with rabbit anti-Myc (Santa Cruz) or control IgG antibodies and assayed by qPCR for enrichment of the indicated loci. Similar results were obtained with mouse anti-Myc antibodies (not shown). **C)** Activity of CG5033- and Uhg1-luciferase reporters in S2 cells upon Myc-overexpression or Myc-knockdown. As a control for dsRNA against Myc (Myc-kd), dsRNA against GFP was used (ctr-kd); for Myc-overexpression tubulin-GAL4 was co-transfected with UAS-Myc, and control samples were transfected with tubulin-GAL4 and UAS-T (empty vector). Error bars show standard deviations from six transfections. *P <0.01; **P <0.005 (Student’s two-tailed t-test).

Seven such genes have been identified in *Drosophila*, numbered 1 to 5, 7, 8 (the gene formerly called Uhg6 is now known as Nop60B); the existence of an additional Uhg gene is suggested by our data (see below). These genes do not contain recognizable open reading frames, and no translation products have been detected (data not shown; see [37]). Instead, they are dedicated to the generation of snoRNAs: Uhg genes contain up to 16 introns, each of which codes for a single snoRNA – 48 snoRNAs for the previously described Uhg genes combined (only two introns do not code for any snoRNA). All of these Uhg (exonic) transcripts are downregulated upon Myc-depletion in the RNAseq data set. qPCR on biologically independent samples confirmed this downregulation (Figure 2B). Since all but three of these Uhg-hosted snoRNAs are shorter than 125 nucleotides and, therefore, not contained in our RNAseq data set, we investigated several of these transcripts by qPCR and found that they were also downregulated (Figure 2B). Such a downregulation can also be seen on Northern blots, which further confirms that the mature snoRNAs are affected by Myc depletion and not just their immature precursors (Figure 2C shows three snoRNAs that could be detected in S2 cells; see legend). Myc is not only required for the full expression of these Uhg-genes and snoRNAs hosted within their exons, but transient overexpression of Myc is also sufficient to increase their expression to supraphysiological levels (Figure 2D). Thus, Myc controls the expression of many snoRNAs independently of their stability or the induction of their protein-coding host genes – and it does so by direct binding to four of the seven Uhg genes. For Uhg-genes 1, 2 and 5 the Myc peaks are centered on consensus E-boxes, for Uhg4 the Myc peak is located in the vicinity of an E-box (Figure 3A, Additional file 2: Figure S6). In addition, Myc binds to a cluster of three snoRNA genes on chromosome 2R, that are arranged in tandem and flanked by a single strongly Myc-bound consensus E-box (Additional file 2: Figure S6B, labeled ‘2R-cluster’). Since only seven other snoRNA genes are independently transcribed in *Drosophila*, we surmise that this cluster might constitute a novel Uhg locus. The binding of Myc to Uhg sequences was confirmed by ChIPs on biologically independent samples (Figure 3B). It presumably involves Myc:Max complexes (as would be expected for E-box containing targets), since knockdown of Myc’s dimerization partner Max reduces Myc recruitment to these Uhg promoters (data not shown). To further corroborate Myc’s transcriptional effect on Uhg genes, we cloned a 983 bp genomic DNA fragment, comprising the basal promoter and E-box of the Uhg1 locus, in front of firefly luciferase. This Uhg1 promoter fragment drives strong expression of luciferase upon transfection into S2 cells. RNA-mediated knockdown of Myc reduces relative luciferase expression to a similar extent as that of a reporter driven by the promoter of the previously characterized Myc target CG5033. Conversely, Myc overexpression significantly increases luciferase expression (Figure 3C; see [38]). Similar results were obtained with a luciferase reporter driven by the Uhg5 promoter region (not shown). Note that reporter activities change more moderately after Myc overexpression than after knockdown. This suggests that Myc levels in naïve S2 cells may be sufficiently high to saturate the Myc binding sites in the reporter plasmids; alternatively, essential co-factors of Myc may become limiting in conditions of increased Myc expression.

### Myc control of snoRNA levels in vertebrates

Taken together, these data demonstrate that *Drosophila* Myc directly controls the expression of snoRNAs. To determine whether this activity of Myc is evolutionarily conserved, we re-evaluated ChIPseq and RNA expression data from vertebrate cells. The human genome encodes 745 snoRNAs, 419 of which reside in introns of 232 different host genes [39], including 15 non protein-coding SNHG loci (‘snoRNA host genes’). To address their regulation by vertebrate Myc, we first examined published data from human U2OS cells [13]. When all genes were sorted by the level of Myc binding to their promoters, we observed a significant enrichment for snoRNA host genes among the highly Myc-bound genes (gene set enrichment analysis in Additional file 2: Figure S7A). Thus, as was described for genes involved in ribosome biogenesis and translation [13], snoRNA host genes are bound by the low physiological levels of c-Myc in U2OS cells. These genes are also activated by c-Myc, as can be seen in murine T-cell lymphomas caused by expression of a tet-controllable Myc transgene [40]. Silencing of the Myc transgene in this system leads to tumor regression. This is accompanied by the rapid up- and down-regulation of 2,287 and 922 genes, respectively, as was determined by Agilent microarrays [41]. More than 70% of the latter genes are bound by Myc and, therefore, have been proposed to correspond to direct transcriptional targets. snoRNA host genes are highly enriched among Myc-activated genes (gene set enrichment analysis in Additional file 2: Figure S7B). Together with the ChIPseq data above, this shows that Myc directly controls the expression of these host genes, and it suggests that Myc also globally activates the levels of many intronic snoRNAs.

**Figure 4:**
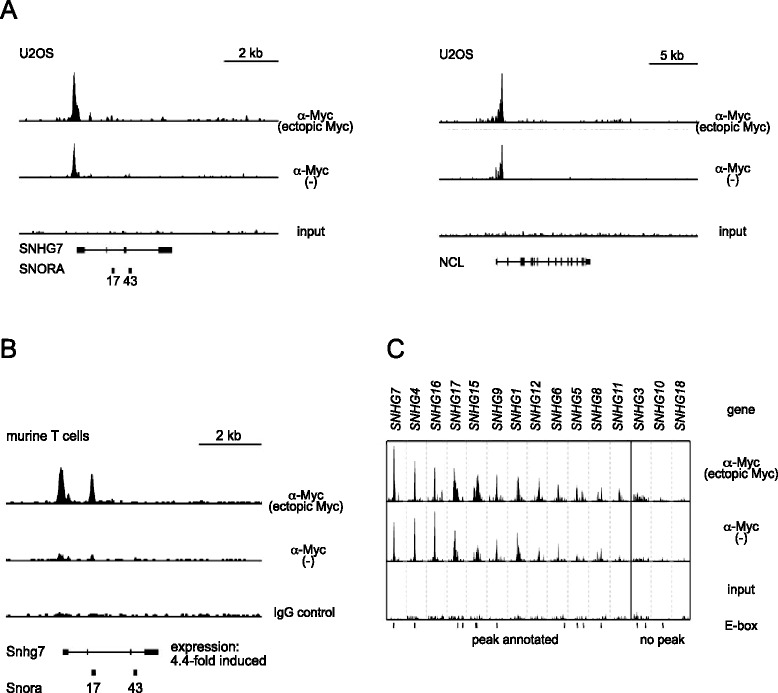
Myc binds to vertebrate non-coding snoRNA host genes (SNHGs). **A)** ChIPseq traces of SNHG7 (left) and NCL (right; a well characterized, strongly Myc-bound gene) in human U2OS cells, both shown at the same vertical scale. **B)** ChIPseq traces of SNHG7 in murine T cells; the expression level of SNHG7 drops by 4.4-fold after silencing of transgenic Myc expression. **C)** ChIPseq traces for all 15 SNHG loci in U2OS cells. Each window is 2 kb wide. Horizontal bars below the input samples denote canonical E-boxes. Samples ‘ectopic Myc’ contain overexpressed Myc, samples ‘-‘ only endogenous Myc; overexpression of Myc was induced (A; tet-on system) or repressed (B; tet-off system) by the addition of doxycyclin. ChIPseq, chromatin immunoprecipitation sequencing; snoRNA, small nucleolar RNA.

The impact of vertebrate Myc on snoRNA synthesis can also be observed at the so-called SNHG loci – genes that are dedicated to the production of snoRNAs, analogously to the *Drosophila* Uhg genes. As an example, the SNHG7 promoter is strongly bound by the low levels of endogenous c-Myc in U2OS cells (Figure 4A, center lane in the left panel); this association is at least as strong as that between Myc and its well-established target Nucleolin ([42]; Figure 4A, center lane in the right panel). Induction of ectopic Myc expression increases the binding to both SNHG7 and Nucleolin further (Figure 4A; top lanes), but only slightly augments the expression of these genes (by 1.2 and 1.3 fold, respectively), probably because they are already maximally transactivated by endogenous Myc [13]. In contrast, in murine T cells expression of endogenous Myc and binding to the Snhg7 promoter are virtually undetectable (see Figure 4B, center lane). In these cells, derepression of transgenically expressed Myc triggers strong association with the Snhg7 promoter, and concomitantly induces Snhg7 mRNA expression by 4.4-fold (Figure 4B, top row). Importantly, Myc is not only recruited to SNHG7, but to the majority of SNHG promoters in murine and human cells, most often to positions containing canonical E-boxes (Figure 4C and data not shown; [41]). These data show that the control of non-coding snoRNA host genes, in particular, and presumably of snoRNA levels, in general, are an evolutionarily conserved function of Myc proteins.

### Uhg-genes promote growth *in vivo* in *Drosophila*

**Figure 5:**
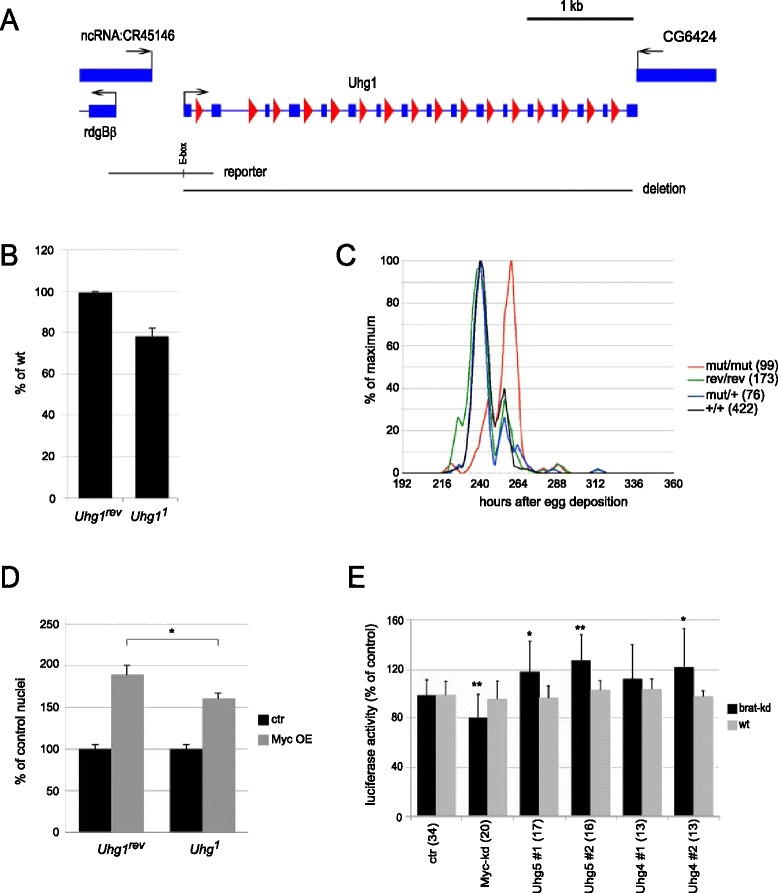
Biological effects of Uhg genes. **A)** Uhg1 locus with adjacent genes. Black arrows indicate direction of transcription, blue rectangles and lines correspond to exons and introns, respectively, red triangles show snoRNAs. Below are indicated the genomic regions used for the luciferase reporter (with the single E-box shown as a vertical line) and the extent of the deletion in the *Uhg1* ^*1*^ null allele. **B)** Amino acid incorporation rates in wandering larvae. Ratios (*Uhg1* ^*1*^/*Uhg1* ^*rev*^) and standard deviations are shown for three biological replicates each with ten larvae for each genotype. Statistical significance of the difference *P* = 0.01 (Student’s two-tailed t-test). **C)** Average time from egg deposition to adult eclosion for male flies; numbers of analyzed flies are indicated in parentheses. ‘mut’ corresponds to ‘*Uhg1* ^*1*^’, ‘rev’ to ‘*Uhg1* ^*rev*^’, ‘+’ to the standard lab strain ‘y w’. **D)** Average area of salivary gland nuclei overexpressing Myc (‘Myc OE’) relative to neighboring nuclei without Myc overexpression (‘ctr’). Each bar represents 39 to 52 nuclei from 10 separate salivary glands; error bars indicate SEM. **E)** Luciferase assays from single adult males overexpressing the indicated Uhg-transgenes under brat-knockdown (brat-kd) or brat-wildtype (ctr) conditions in type II neuroblast lineages. #1 and #2 correspond to independent transgenes; numbers in parentheses indicate the numbers of individually assayed flies (originating from two to ten separate experiments). Genotypes: in addition to the indicated UAS-Uhg transgene, the flies carried ‘worniu-GAL4 asense-GAL80/+; UAS-brat-(inverted repeat) UAS-Luciferase/+’. Difference to control is <0.05 (*) and <0.005 (**), respectively (Student’s two-tailed t-test). SEM, standard error of the mean; snoRNA, small nucleolar RNA.

To address the importance of snoRNAs for growth, we focused on the Uhg1 locus since it encodes 16 snoRNAs, but no other potentially growth-relevant transcripts. Using imprecise excision of a P-element we generated a null allele that eliminates the coding regions for all snoRNAs, but does not affect the neighboring genes (*Uhg1*^*1*^; Figure 5A and Additional file 2: Figure S8A). This mutation is expected to affect 2’-O ribose-methylation of 18S- and 28S-rRNA at five positions that are exclusively targeted by Uhg1-snoRNAs [43], and as a consequence ribosomal assembly and/or activity. Interestingly, 66% of the resulting *Uhg1*^*1*^/*Uhg1*^*1*^ mutant females and 50% of the males survive to adulthood (n = 131 and 99, respectively). These adults are not significantly lighter than control animals (homozygotes for the wildtype reversion allele *Uhg1*^*rev*^ that was obtained by precise excision; Additional file 2: Figure S8B). Similarly, *Uhg1*^*1*^ mutant wandering larvae contain at least as much protein as control animals (Additional file 2: Figure S8C). However, the *Uhg1*^*1*^ grow at a significantly slower rate (as inferred from their rate of incorporating radiolabeled amino acids; Figure 5B) and accordingly reach corresponding developmental stages later than wild type flies – adult eclosion is delayed by about 12 hours in *Uhg1*^*1*^ mutants (Figure 5C, Additional file 2: Figure S8D). In addition, *Uhg1*^*1*^ females, but not males, show a strongly reduced fertility (not shown). Both such a developmental delay and female-specific sterility are typical manifestations of mutations in pathways affecting growth, and they have been observed for hypomorphic mutations in Myc [4]. Consistent with a contribution of Uhg1 to Myc-dependent growth, the documented ability of Myc to promote nuclear growth is blunted in Uhg1 mutant salivary gland cells (Figure 5D; compare the relative size of Myc-overexpressing nuclei in wild type versus Uhg1 mutant salivary glands). Together, these data demonstrate that Uhg1 is required for protein synthesis and growth. Nevertheless, the complete absence of Uhg1 has comparatively mild consequences, and in some tissues Uhg1 seems not to be limiting for growth and proliferation. We did not observe any impact of the *Uhg1*^*1*^ mutation on the area of imaginal disc cell clones overexpressing Myc (not shown). Similarly, larval brain tumors were not obviously influenced by the complete loss of Uhg1: knockdown of the tumor suppressor *brat* in type II neuroblast stem cell lineages leads to tumors that critically depend on Myc [44]. However, homozygosity for *Uhg1*^*1*^ in such a setting did not reduce tumor mass, nor did it prevent the *brat* knock-down induced reversion of intermediate progenitor cells to supernumerary neuroblasts (not shown). It is possible, though, that the extension of larval development that is caused by the loss of Uhg1, masks a potential reduction in tumor growth rate. As a complementary approach that does not suffer from confounding effects on developmental timing, we therefore investigated the consequences of excessive Uhg levels. We generated various Uhg transgenes and expressed them in different settings. Overexpression of Uhg genes on their own did not increase the size of normal diploid or polyploid cells, nor did this treatment affect the size of cells co-expressing Myc (data not shown). However, independent transgenes for Uhg4 and Uhg5 increased the size of brat-knockdown induced brain tumors, as assayed with a recently described luciferase-based system (Figure 5E; [5]). Thus, overexpression of at least two different Uhg-genes promotes the growth of tumors.

## Discussion

**Figure 6:**
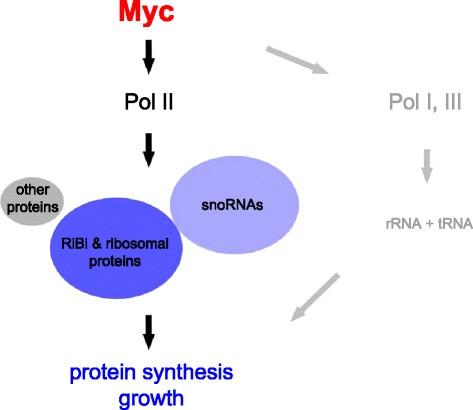
Schematic of Myc function in *Drosophila*. The majority of RNA Polymerase II transcribed direct Myc targets (defined as genes that are bound and activated by Myc) control ribosome biogenesis (RiBi genes) or code for ribosomal proteins. The area of each group is proportional to the number of genes it contains; intronic snoRNAs are counted as direct Myc targets if their host genes are direct Myc targets. Previously, Myc has been shown to activate RNA Polymerases I and III and hence the transcription of rRNAs and tRNAs. Thus, the major function of *Drosophila* Myc resides in the control of protein synthesis. snoRNA, small nucleolar RNA.

In this study, we combine RNAseq and ChIPseq approaches to identify a core set of direct Myc targets in *Drosophila* S2 cells. We find that *Drosophila* Myc controls less than 500 genes, substantially fewer than have been proposed to be controlled by vertebrate Myc [10-13,45]. The overwhelming part of *Drosophila* Myc targets control ribosome biogenesis and protein synthesis, which, therefore, constitute the core and presumably primordial function of Myc proteins. Among these core targets we find an entire novel gene class that has previously not been recognized as Myc targets: the non protein-coding snoRNAs. Some of Myc’s effect on snoRNA levels may be indirect (via the activation of their host genes or by affecting their stability), but a sizable fraction of snoRNAs encoded in Uhg genes is directly transcriptionally activated by Myc (Figure 6).

snoRNAs mostly fall into either the box H/ACA or the box C/D class, depending on the structural elements they contain (reviewed by [23]). Upon binding to specific protein partners they form snoRNPs that process rRNAs (by catalyzing pseudouridylation, methylation and cleavage) and contribute to their folding, thereby playing an important role in ribosome biogenesis. In addition, some snoRNAs have been shown to regulate alternative splicing [46], affect RNA editing [47], mediate apoptosis in response to lipotoxic stress [48], serve as progenitors of miRNAs (reviewed by [49]), and they may contribute to the maintenance of open chromatin structure [50]. Most animal snoRNAs are encoded in introns of other genes, many of which code for ribosome- and translation-related proteins [34]. This arrangement is thought to help adapt snoRNA production to a cell’s biosynthetic needs. Here, we identify a new means by which the balanced synthesis of the different components of snoRNP complexes is achieved. The protein partners of snoRNAs are well established direct transcriptional targets of Myc, that is, the box C/D snoRNP components Fibrillarin, Nop56, Nop58/Nop5, 15.5K/hoi-polloi, and the box H/ACA snoRNP components GAR1, Dyskerin/Nop60B, NHP2, NOP10/CG7637. Moreover, several factors required for snoRNP assembly or localization are direct Myc targets (for example, Nopp140, U3-55K, DDX18/pitchoune, DDX51/Dbp73D) or show close genetic and physical interaction with Myc (Tip48/reptin, Tip49/pontin) (Additional file 1: Table S4 and reviews by [3,23]). Thus, a single transcription factor, Myc, controls all components that make up snoRNP particles, as well as several factors that contribute to their function, thereby acting as a master regulator of snoRNP production. At the same time, the production of snoRNPs and rRNA modifications constitute the core function of Myc, as 48% of its mostly highly controlled target genes are dedicated to this task. Our data further show that this control of snoRNA levels by Myc is biologically relevant. The loss of Uhg1-encoded snoRNAs impairs normal growth, whereas overexpression of a subset of Uhg genes promotes tumor growth in a Myc-dependent brain tumor system.

While our studies have focused on *Drosophila*, we also found that Myc’s control of snoRNA host genes is conserved in vertebrates. Indeed, recent publications provide support for Myc’s role in snoRNA expression. A bioinformatic analysis of 131 intergenic human snoRNA promoters found an enrichment of E-boxes, that is, potential Myc binding sites [39]. This suggests that, in addition to intronic snoRNAs (our data), independently transcribed snoRNAs might be activated by direct Myc binding. Moreover, several snoRNAs were found to be significantly overexpressed in neuroblastomas with high-level N-Myc expression (that is, N-Myc amplification), as well as in cultured cell lines in response to N-Myc activation [51]. This observation raises the possibility that snoRNAs contribute to the growth of N-Myc dependent tumors, analogously to what we observed upon overexpression of Uhg genes in *Drosophila* type II neuroblast tumors. Several publications point to a possible role for snoRNA deregulation in other cancers as well (for reviews see [52,53]). Thus, the levels of snoRNAs are elevated in non-small-cell lung cancer (NSCLC), acute leukemias, and metastatic prostate cancer [54-56], and individual snoRNAs have been shown to contribute to cellular proliferation and/or transformation (SNORD114-1 in K562 cells [55], SNORA42 in breast cancer [57]). On the other hand, certain snoRNAs were also shown to have a negative impact on proliferation (snoRNA U50 in prostate cancer [58]), breast cancer [59], and some tumors display reduced snoRNA levels (multiple myeloma and secondary plasma cell leukemia: [60]).

## Conclusions

In transformed vertebrate cells, the oncogene Myc is thought to act as a general transcriptional amplifier. In contrast, our present study shows that at physiological levels in *Drosophila* cells, Myc directly binds and controls only a limited number of transcriptional targets – less than 500. In addition to previously described Myc targets that contribute to ribosome biogenesis and protein translation, we find that Myc controls the levels of all snoRNAs. Most of these small RNAs function as part of ribonucleoproteins to post-transcriptionally modify rRNAs, which then presumably affects ribosome activity. Indeed, several observations in vertebrate tumors (as well as our experiments in *Drosophila*) suggest a correlation between the levels of certain snoRNAs and the rate of normal and/or pathological growth. It remains to be seen whether this reflects a global alteration of protein synthesis or differential effects on specific proteins. The accessibility of the *Drosophila* system, both for loss-of-function and overexpression approaches, raises the possibility of experimentally addressing these issues in the future. In short, our data highlight the non-coding snoRNAs as a novel and evolutionarily conserved class of direct Myc targets that are likely to play an important role both in normal development, as well as in Myc-dependent pathological growth.

## Methods

### Generation of mutant flies, transgenes and reporters

The allele *Uhg1*^*1*^ (null mutant) and *Uhg1*^*rev*^ (wild type revertant) were generated by mobilization of P{w[+mC] = EP}Uhg1[G11659] (Bloomington Stock Center number B-28084, Indiana University; Bloomington, IN, USA), inserted after position chr2R:13’586’605 (coordinates throughout refer to genome release 5) in Uhg1 exon 1. *Uhg1*^*1*^ carries a deletion of nucleotides chr2R:13’586’606 to 13’590’803 (first to last exon of Uhg1).

UAS-Uhg transgenes were generated in a ‘y w’ background by cloning appropriate PCR fragments (Additional file 1: Table S5) into pUAS-T, followed by sequence verification and standard transgenesis procedures.

Additional flies: wor-GAL4 ase-GAL80/CyO; UAS-brat-IR UAS-FLuc/TM3, Sb Ser tubulin-GAL80[ts] [5], actin-FRT-CD2-FRT-GAL4 hs-FLP (H.Stocker, ETH Zürich, Switzerland), UAS-vito::GFP [61] (Bloomington Stock Center).

### *In vivo* analysis

To measure the time from egg deposition to adult eclosion, timed egg lays (5 to 14 hours) were performed in 10 to 50 culture vials per genotype (‘y w; *Uhg1*^*1*^/CyO, y + ’, ‘y w; *Uhg1*^*rev*^’, ‘y w’, ‘y w; *Uhg1*^*1*^/CyO,y+’ x ‘y w’), so as to avoid overcrowding. Eclosion was monitored two to three times a day. Developmental times were combined using a weighted 10-hour floating window (perl script available upon request).

For weighing flies, one to four day old flies were dried for 20 minutes at 95° (first for 10 minutes with a closed, then with an opened lid) and then stored at room temperature. Before weighing on a Mettler UMT5 Comparator scale (Mettler Toledo, Giessen, Germany), the flies were allowed to equilibrate with ambient atmosphere for at least 30 minutes.

For clonal overexpression of Myc, the genotypes ‘y w actin-FRT-CD2-FRT-GAL4 hs-FLP/y w; *Uhg1*^*1*^/*Uhg1*^*1*^; UAS-vito::GFP/UAS-Myc UAS-GFP’ and ‘y w actin-FRT-CD2-FRT-GAL4 hs-FLP/y w; (Sp or CyO,y+)/+; UAS-vito::GFP/UAS-Myc UAS-GFP’ were analyzed. At two to three days of development, clones were induced by an 8- to 15-minute incubation in a 37° water bath; 48 hours later, wandering larvae were collected, fixed for 20 minutes at room temperature in 4% paraformaldehyde/PBS containing 0.1% Tween-20, stained with 10 μg/ml Hoechst 33258 (Sigma Aldrich, St. Louis, MO, USA) and 2 u/μl Alexa Fluor 546 Phalloidin (Molecular Probes, Life technologies; Carlsbad, CA, USA), and destained with PBS-Tween before mounting in Vectashield Mounting Medium (Biozol Diagnostica, Eching, Germany). Images were recorded on a Nikon Eclipse Ti-E confocal microscope, using a 20 × lens.

To determine protein translation activity in wandering larvae, 750 μl Ringer’s solution containing 15 μCi/ml ^3^H-labeled amino acid mix (Hartmann Analytic, Braunschweig, Germany) were added to 10 fully inverted larvae and placed on a rotating wheel for one hour at room temperature. The supernatant was then decanted and the carcasses were washed twice with cold Ringer’s solution before they were crushed in 350 μl cell lysis buffer (100 mM Tris-Cl (pH 8.0), 100 mM NaCl, 0.5% Triton X-100) with a pestle. Samples were incubated for 10 minutes on ice with periodic vortexing, followed by a 2 minute centrifugation. A total of 250 μl of the aqueous lysate was mixed with 15 μl suspended Strataclean Resin (Stratagene, Agilent Technologies; Santa Clara, CA, USA) and allowed to rest for five minutes. After removing the supernatant, resins were washed with Ringer’s solution and transferred to 3 ml scintillation buffer, rested for 30 minutes, and then counted for 1 minute per vial. The remaining aqueous lysate was processed for protein quantification [62].

Type II neuroblast tumors were induced by knocking down the tumor suppressor *brat* specifically in these cells with the driver system ‘worniu-GAL4 asense-GAL80.’ Co-expression of firefly luciferase allowed the quantitative determination of tumor mass [5]. Appropriate male adults (at most 12 hours old) were collected and frozen at −80° until use. Upon thawing, individual flies were lysed in Passive Lysis Buffer (Dual Luciferase Reporter Assay System; Promega, Madison, WI, USA) in a Bullet Blender homogenizer (Next Advance, Averill Park, NY, USA) and processed for luminometry in a Glomax luminometer (Promega).

### Cell culture and RNA interference

*Drosophila* S2 and Kc167 cells were cultivated at 25°C in Schneider’s Insect Medium (Sigma) including 10% fetal bovine serum (Pan™ Biotech) and 1% penicillin/streptomycin (Sigma). For RNAi experiments, cells were plated out and washed once in serum free medium. dsRNA against Myc, or GFP for control, was added directly to the cells kept in 1/3 volume of serum free medium. After 30 minutes 2/3 volume of full medium was added. A total of 2 μg of dsRNA were added per 10^6^ cells. Cells were harvested 24 hours after RNA addition.

### Western blotting

S2 cells were lysed in lysis buffer (150 mM NaCl, 50 mM Tris (pH 8.0), 5 mM ethylenediaminetetraacetic acid (EDTA) (pH 8.0), 1% Nonidet-P40), mixed with an equal amount of Laemmli buffer (containing 20% β − mercaptoethanol) and an extract from 1.25 × 10^6^ cells was separated on a 10% SDS-PAGE. Proteins were transferred to a nitrocellulose membrane and incubated with appropriate antibodies (mouse anti-Myc (described in [25]), rabbit anti-Myc [26], rabbit anti-Myc (Santa Cruz, Dallas, TX, USA), mouse anti-α-Tubulin (Sigma)).

### RNAseq

Total RNA was isolated from *Drosophila* S2 cells using Trizol (Invitrogen, Life Technologies; Carlsbad, CA, USA) and purified with miRNeasy (Qiagen, Venlo, Netherlands), followed by on-column DNase treatment to eliminate genomic DNA, all according to the manufacturer’s instructions. Samples were treated once with RiboMinus™ (Life Technologies, Carlsbad, CA, USA) to selectively deplete ribosomal RNA (rRNA). Sequencing libraries were prepared with the NEBnext® mRNA Library Prep Master Mix set for Illumina (E6100S/L) following the instruction manual. Briefly, ribominus-treated RNA was fragmented, first and second cDNA strands were synthesized, and the resulting duplex was end-repaired, ligated to NEBnext adaptors and gel purified with the Qiagen gel extraction kit selecting 200 bp fragments. cDNA was then amplified with 15 cycles of PCR and the resulting library was subjected to Illumina GAIIx sequencing. For both Myc-knockdown (experimental) and GFP-knockdown (control) samples, three biologically independent replicates were prepared and analyzed. Quality and quantity of RNAs and resulting cDNAs were assessed at several steps of the procedure through an Experion™ Automated Electrophoresis System (Bio-Rad, Hercules, CA, USA).

Sequence data were processed through the bioinformatics pipeline OLB_v1.9, then through a Perl script to eliminate reads with perfect matches to *Drosophila* rRNA. The non-rRNA reads were mapped to the *Drosophila* genome release 5 with bowtie-0.12.7. (between 2,082,423 and 14,466,659 mapped reads per condition and repeat), converted from sam to bam format with SAMtools, and statistically analyzed with the BioconductoR work package. For subsequent analysis we normalized the total read number from each RNAseq experiment to 1,000,000. Only genes with ≥10 reads combined in all six samples and with predicted transcript sizes ≥ 125 nt were kept for final analysis (for a total of 8,019 genes). RNAseq and ChIPseq data are available in the ArrayExpress database [63] under accession number E-MTAB-3209.

### ChIPseq

For chromatin immunoprecipitation (ChIP), cells were cross-linked with 1% formaldehyde at 37°C for 10 minutes and the reaction was stopped with 50 mM glycine. Cells were lysed and nuclei were resuspended in radioimmunoprecipitation assay (RIPA) buffer. Sonication with a Branson sonifier was carried out until the majority of fragments showed nucleosomal size. Cells were immunoprecipitated with Myc antibody (mouse α-Myc, rabbit α-Myc, Santa Cruz rabbit α-Myc) or control IgG from mouse or rabbit (Sigma) which were coupled to Protein A/G-dynabeads (Invitrogen). DNA was purified with phenol-chloroform after elution of the bound chromatin with 1% SDS and reversion of the crosslink.

ChIP DNA was end repaired and A tailed. Illumina adaptors were ligated to the ChIP DNA fragments. Fractions with a size of 175 to 225 bp were cut out from a 2% agarose gel, extracted by Qiagen gel extraction kit and enriched by 18 cycles of PCR amplification. The library-size was controlled with the Experion-system (BioRad) and quantified using a picogreen assay. The library was sequenced on a Illumna GAIIx sequencer.

Sequence data were processed with OLB_v1.9 and mapped to the *Drosophila* genome release 5 with bowtie-0.12.7. For each condition, we obtained between 12,569,801 and 33,927,814 reads, for which between 47% and 75% aligned to a single position in the *Drosophila* genome and were further considered, 2% to 31% did not align anywhere, and the remainder showed multiple alignments (corresponding typically to transposons and heterochromatic regions). Peaks were identified with MACS14, using the same number of reads with single alignment for all conditions (6,874,000) and the default settings (with the switch ‘-g dm’). Number of called peaks for the different conditions: 260 and 27 (mouse α-Myc and non-immune mouse IgG in naïve S2 cells), 22 and 24 (mouse α-Myc and non-immune mouse IgG in Myc-depleted S2 cells), 263 and 31 (rabbit α-Myc and non-immune rabbit IgG in S2 cells), 308 and 187 (rabbit α-Myc and non-immune rabbit IgG in Kc167 cells). Subsequently, we eliminated from the Myc-ChIP lists all unmapped peaks (mostly assigned to chromosome ‘Uextra’; 3 to 17 peaks per condition) and all peaks that were called in any of the ChIPs with non-immune IgGs or in Myc-ChIPs from Myc-depleted S2 cells, resulting in 240 (mouse α-Myc in S2 cells; 240 with FDR < 10%), 243 (rabbit α-Myc in S2 cells; 98 with FDR <10%), 279 peaks (rabbit α-Myc in Kc167 cells; 21 with FDR < 10%). Additional file 1: Table S1 lists all 265 peaks that are significant (FDR <10%) in at least one condition, and includes non-significant peaks (FDR ≥10%) if they overlap a significant peak.

### qPCR of S2 cells

For ‘standard’ qPCR, total RNA was isolated from Myc and control depleted *Drosophila* S2 cells as described for RNAseq. The miScript II RT Kit (Qiagen) was used to generate cDNA which allows the conversion of all RNA species. cDNA was analyzed as described for ChIP DNA, using the ΔΔCT method for evaluation of the results. Every qRT-PCR was performed in triplicate for at least two biologically independent samples. The averages of two or three of the reference genes rab6, snm158 and α-Tubulin were set to 100%, since these genes were found to be unaffected by Myc-knockdown in our RNAseq, as well as earlier experiments.

### Northern blot

Total RNA was extracted from *Drosophila* S2 cells as described above. A total of 20 μg RNA was loaded per lane on a 10% acrylamide 8 M urea gel and then transferred to a Hybond N+ nylon membrane (GE Healthcare, Chalfont St Giles, Great Britain (Frankfurt)). After UV crosslinking at 254 nm the membrane was pre-hybridized in 10 ml Church buffer (1 mM EDTA pH 8.0, 0.17% phosphoric acid, 0.5 M Na_2_HPO_4_, 7% SDS) for one hour at 62°C. DNA oligonucleotide probes were 5′ end-labeled with γ-^32^P-ATP using T4 polynucleotide kinase (NEB) and hybridized overnight in Church buffer at 62°C. The blots were washed in 2 × SSC (300 mM NaCl, 30 mM sodium citrate) and 0.2 × SSC for 30 minutes at 62°C, dried, exposed and developed on a Typhoon 9200 phosphorimager (GE healthcare). Quantification was performed using ImageJ software.

### Uhg1 reporter analysis

A 983 bp genomic fragment (chr2R: 13,585,896 to chr2R: 13,586,878) encompassing the TSS of Uhg1 (chr2R:13,586,602) and the E-box at chr2R:13,586,588 was cloned in front of the firefly luciferase coding sequence and the SV40 polyadenylation signal, yielding pGL-Uhg1WT. For luciferase assays, 1.3 × 10^6^ S2 cells were plated into each well of a 24-well plate and transfected with 0.2 μg of reporter plasmid (pGL-Uhg1WT or pGL-CG5033WT) and 200 ng of reference plasmid pRL-CG5033ΔE, using Effectene (Qiagen) according to the manufacturer’s instructions (for CG5033-plasmids, dsRNA and assay protocol, see [38]). Where indicated, 30 ng of dsRNA against Myc or GFP, or 200 ng pTub-GAL4 plus 200 ng pUAS-Myc or pUAS-T, were included in the transfection mix. One day after the transfection, cells were harvested, lysed and firefly and Renilla luciferase activities were measured using the Dual Luciferase Assay System (Promega) in a Glomax luminometer. Each experiment was carried out at least twice (on separate days), and every transfection within each experiment was done in triplicate.

### Further bioinformatic analysis

To calculate distances of the 265 peaks to the nearest transcription start sites (TSS) we used closestBed from the Bedtools suite v.2.17.0. and the *Drosophila* genome annotation FlyBase fb_2013_05. To assign genes to mapped ChIP peaks, we used intersectBed and closestBed and subsequently manually pruned the gene lists. For ChIPseq peaks overlapping at least one gene with statistically significant expression change upon Myc depletion (*P* < 0.05), only transcriptionally affected genes were retained. When a peak mapped within 100 bp of the TSS of a transcriptionally unaffected gene, other transcriptionally unaffected genes with TSS at more than 300 bp distance from the peak were eliminated (20 instances). For intronic snoRNAs, only the host genes were retained.

For the analysis of peaks from Yang *et al*. [27] we used the data published by these authors and the program intersectBed (bedtools v.2.17.0.) to determine the ratio of mouse α-Myc ChIPseq reads from naïve S2 cells versus Myc-depleted S2 cells for each of the 3,993 peaks. In total, 1,936 regions with a ratio ‘naïve/Myc-depleted’ ≤ 1.2 that did not overlap any of our peaks were retained and sorted by the number of reads that were recorded by Yang *et al*. From this sorted list we selected every 50^th^ peak (for a total of 20 peaks), starting with the region with the highest number of reads in the Yang analysis, and designed primers covering the summit coordinate indicated by Yang *et al*. (one region was omitted since no acceptable primers could be generated, and two primer pairs did not function, that is, did not produce the expected product).

For GO term analysis we used the online resource GOrilla [64] and all *Drosophila* genes according to FlyBase annotation fb_2013_05 as the background set. Identification of binding sites was carried out with the online resource MEME-ChIP [65]. Gene Set Enrichment Analysis was performed with the GSEA software [66]. ChIPseq reads were visualized with the IGB browser [67]. Venn diagrams were drawn with the help of eulerAPE v.1.0 [68].

## Additional files

Additional file 1: Table S1. Myc-bound genomic sequences as determined by ChIPseq. **Table S2.** Genes associated with Myc-binding sites. **Table S3.** Myc-dependent regulation of gene expression in S2 cells. **Table S4.** Directly Myc-activated genes, sorted by biological category. Corresponds to **Table** 1 **.** **Table S5.** Sequences of PCR primers that were used for quantitative reverse transcription, ChIP-PCR, subcloning, and Northern blots. Additional file 2:
**ChIP signals of Yang peaks from naïve and Myc-depleted S2 cells.** **Figure S2.** Efficiency of Myc-depletion in S2 cells used for RNAseq. **Figure S3.** Overlap of Myc-target lists from different experimental approaches. **Figure S4.** Effect of Myc-depletion on the expression levels of Myc-bound genes. **Figure S5.** Relative expression levels of snoRNAs and their host genes. **Figure S6.** ChIPseq profiles for Uhg2, Uhg4, Uhg5 and a novel snoRNA cluster. **Figure S7.** Myc binding and transactivation of snoRNA host genes in vertebrates. **Figure S8.** Characterization of *Uhg1* ^*1*^ mutants.
