# Supplementary material for: snoRNAs are a novel class of biologically relevant Myc targets
Source: BMC Biol. 2015 Apr 16;13:25. doi: 10.1186/s12915-015-0132-6 (PMC4430873; doi:10.1186/s12915-015-0132-6)
Supplement: Additional file 2: — ChIP signals of Yang peaks from naïve and Myc-depleted S2 cells. Figure S2. Efficiency of Myc-depletion in S2 cells used for RNAseq. Figure S3. Overlap of Myc-target lists from different experimental approaches. Figure S4. Effect of Myc-depletion on the expression levels of Myc-bound genes. Figure S5. Relative expression levels of snoRNAs and their host genes. Figure S6. ChIPseq profiles for Uhg2, Uhg4, Uhg5 and a novel snoRNA cluster. Figure S7. Myc binding and transactivation of snoRNA host genes in vertebrates. Figure S8. Characterization of Uhg1 1 mutants. [file 12915_2015_132_MOESM2_ESM.pdf]

Figure Legends

Figure S1. ChIP signals of Yang peaks (see Methods) from naïve and Myc-depleted S2 cells. *A*, ChIPseq traces obtained with the mouse α-Myc antibody or with mouse control IgG. Peak numbers correspond to those in panel B. Note that the indicated peaks are not eliminated by Myc-depletion (compare with Figure 1A) and are also obtained with control IgGs. *B*, ChIP-qPCR after immunoprecipitation with the commercial rabbit α-Myc antibody or with rabbit control IgG. Inset shows the efficiency of Myc-depletion in S2 cells. Asterisk: principal Myc band at 107 kD; arrowhead: band at ≈130 kD that is recognized by both antisera and reduced upon Myc knockdown, probably corresponding to post-translationally modified Myc; arrow: band that is only observed with the Santa Cruz antibody and that is not affected by Myc knock-down. Markers (in kD) are shown on the left.

Figure S2. Efficiency of Myc-depletion in S2 cells used for RNAseq. In three biologically independent experiments, dsRNA directed against Myc or GFP was transfected into S2 cells and cells were harvested 24 hours later. Part of each sample was processed for transcriptome analysis. The remainder was analysed by Western blotting with mouse anti-Myc antibody (top) and mouse anti-α-Tub84B (bottom).

Figure S3. Overlap of Myc-target lists from different experimental approaches. Number of genes that are recognized as significantly Myc-activated (*panel A*) or –repressed (*panel B*) 24 hours after Myc-knockdown in S2 cells (this study; RNAseq in triplicate; 212 genes), 4 days after Myc-knockdown in S2 cells [1; Affymetrix microarrays; 472 genes], 7 or 14 hours after Myc-overexpression in whole larvae [2; spotted microarrays in 4 or 5 copies; 438 genes]. Numbers in red italics between two datasets indicate the probability of a chance overlap (hypergeometric distribution). As expected, the different gene lists show a statistically highly significant overlap – despite the substantial differences between the underlying experimental setups. Thus, experiment 1 assayed transcriptional changes 4 days after Myc knockdown

(which is likely to reveal secondary consequences of the Myc knockdown as well), and experiment 2 involved Myc overexpression rather than knockdown, and it interrogated the complex mixture of cell types contained in whole larvae. Furthermore, microarray technologies (such as used in experiments 1 and 2) have been documented to provide somewhat different output than RNAseq (such as used in experiment 3) [e.g. 3].

Figure S4. Effect of Myc-depletion on the expression levels of Myc-bound genes. *A*, genes bound by Myc in ChIPseq are ordered by their *significance of misexpression* (p-value). The green and red curves show running sums of genes that are down- and up-regulated, respectively, after Myc-depletion; e.g. at a significance cutoff $p \leq 0.20$ 8 genes are down-regulated (red curve) and 139 genes are upregulated (green curve). *B*, Myc-bound genes are ordered by *relative expression level* upon Myc-depletion; 67% and 133% relative expression levels are indicated by the green and red lines, respectively. A total of 190 of the Myc-bound genes are downregulated, many of them statistically significantly (compare with panel A); a total of 89 genes are upregulated, but most often not statistically significantly (compare with panel A).

Figure S5. Relative expression levels of snoRNAs and their host genes. Each dot corresponds to a single intronic snoRNA of at least 125 nt length. The position on the y-axis indicates its relative expression upon Myc-depletion, and the position on the x-axis the relative expression of its protein-coding host gene (exonic sequence). Uhg loci were excluded from this analysis. Most snoRNAs localize to the lower left quadrant, indicating that both snoRNA and its host gene are downregulated upon Myc-knockdown. The data sets with identical x-values correspond to snoRNAs contained in the same host gene (e.g. Nop60B).

Figure S6. ChIPseq profiles for Uhg2, Uhg4, Uhg5 and a snoRNA cluster at 2R:9’445’583 in S2 cells. Labeling conventions correspond to those in Figure 1A.

Figure S7. Myc binds and transactivates snoRNA host genes in vertebrates. Gene set enrichment analysis [GSEA; 4] was used to illustrate the enrichment of 232 vertebrate snoRNA host genes [hosting a total of 419 intronic snoRNAs; 5] amongst the genes bound by Myc in

human U2OS cells (A) or transactivated by Myc in murine T cells (B). *A*, genes were ordered by the intensity of Myc binding to their promoters, with the most highly Myc-bound genes located on the left (gene 1). Promoters were defined as transcription start sites +/- 500 nt. Relative c-Myc binding was calculated as the number of sequence tags obtained from anti-Myc ChIPseq in uninduced human U2OS cells (expressing only endogenous c-Myc), minus the number of sequence tags from the corresponding input control [7]. *B*, genes were ordered by their expression in untreated T cells (ectopic expression of c-Myc), relative to the expression in doxycycline-treated T cells (no ectopic c-Myc expression) [6]; the most highly Myc-activated genes are found on the right (gene 22'500). NES indicates the normalized enrichment score, p the nominal p-value and q the false discovery rate.

Figure S8. Characterization of *Uhg1*^{1} mutants. *A*, expression of Uhg1 and the neighbouring transcripts in *Uhg1*^{1} mutant wandering larvae. Expression was normalized to the average of the 3 reference genes rab6, α-Tubulin, snm158. The Uhg1 amplification product from mutant larvae shows an aberrant melting curve and migration behaviour on agarose gels and therefore presumably corresponds to a non-specific product. The graph shows the average and standard deviations of three biological replicates. *B,* average dry weight and standard deviations of adult flies of the indicated genotypes (n=21 to 34 per genotype). *C*, protein content of wandering larvae. Ratios (*Uhg1*^{1} : *Uhg1*^{rev}) and standard deviations are shown for 3 biological replicates each with 10 larvae for each genotype. *D*, average time from egg deposition to adult eclosion for female flies; numbers of analysed flies are indicated in parentheses. See Figure 5 for genotypes.

References

1. Bonke M, Turunen M, Sokolova M, Vaharautio A, Kivioja T, Taipale M, Bjorklund M, Taipale J: **Transcriptional networks controlling the cell cycle.** *G3* 2013, **3:**75-90.
2. Grewal SS, Li L, Orian A, Eisenman RN, Edgar BA: **Myc-dependent regulation of ribosomal RNA synthesis during Drosophila development.** *Nat Cell Biol* 2005, **7:**295-302.
3. Guo Y, Sheng Q, Li J, Ye F, Samuels DC, Shyr Y: **Large Scale Comparison of Gene Expression Levels by Microarrays and RNAseq Using TCGA Data.** *PLoS ONE* 2013, **8:**e71462.
4. Subramanian A, Tamayo P, Mootha VK, Mukherjee S, Ebert BL, Gillette MA, Paulovich A, Pomeroy SL, Golub TR, Lander ES, Mesirov JP: **Gene set enrichment analysis: a knowledge-based approach for interpreting genome-wide expression profiles.** *Proceedings of the National Academy of Sciences of the United States of America* 2005, **102:**15545-15550.
5. Li T, Zhou X, Wang X, Zhu D, Zhang Y: **Identification and characterization of human snoRNA core promoters.** *Genomics* 2010, **96:**50-56.
6. Müller J, Samans B, van Riggelen J, Fagà G, Peh K.N R, Wei C-L, Müller H, Amati B, Felsher D, Eilers M: **TGFβ-dependent gene expression shows that senescence correlates with abortive differentiation along several lineages in Myc-induced lymphomas.** *Cell Cycle* 2010, **9:**4622-4626.
7. Walz S, Lorenzin F, Morton J, Wiese KE, von Eyss B, Herold S, Rycak L, Dumay-Odelot H, Karim S, Bartkuhn M, et al: **Activation and repression by oncogenic MYC shape tumour-specific gene expression profiles.** *Nature* 2014, **511:**483-487.

Sup. Figure 1

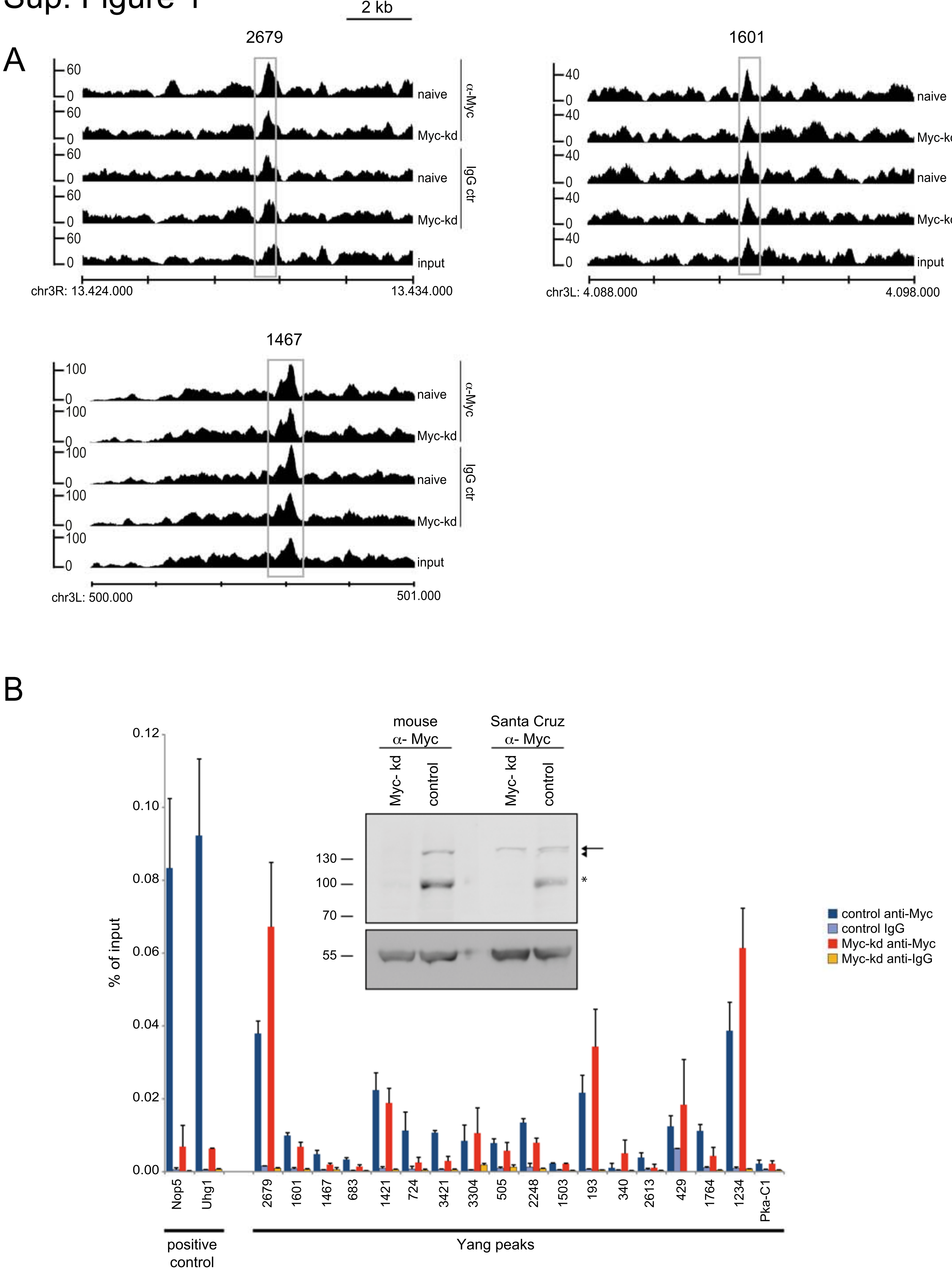

A
2 kb
2679
1601
1467
α-Myc
IgG ctr
naive
Myc-kd
input
chr3R: 13.424.000
13.434.000
chr3L: 4.088.000
4.098.000
chr3L: 500.000
501.000
B
% of input
mouse α- Myc
Santa Cruz α- Myc
Myc- kd
control
130
100
70
55
control anti-Myc
control IgG
Myc-kd anti-Myc
Myc-kd anti-IgG
Nop5
Uhg1
2679
1601
1467
683
1421
724
3421
3304
505
2248
1503
193
340
2613
429
1764
1234
Pka-C1
positive control
Yang peaks

Sup. Figure 2

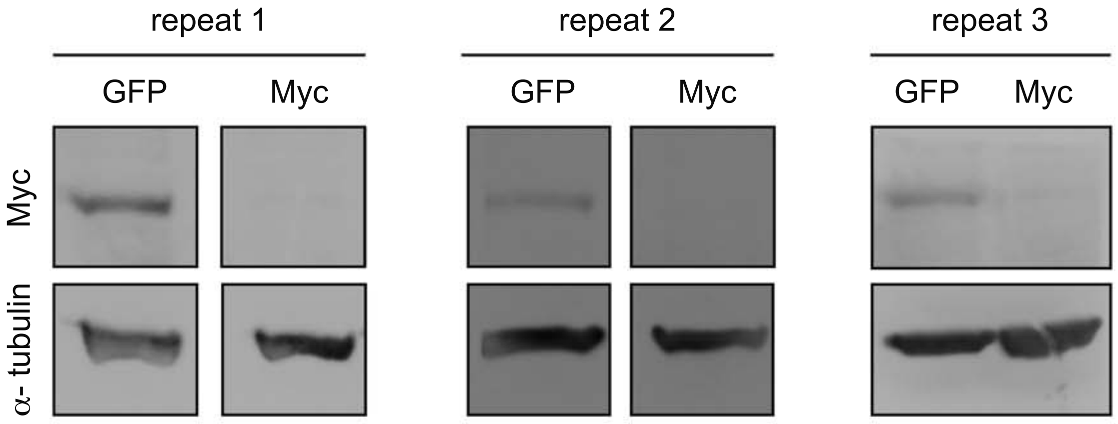

repeat 1
repeat 2
repeat 3
GFP
Myc
GFP
Myc
GFP
Myc
Myc
α- tubulin

Sup. Figure 3

A: genes activated by Myc

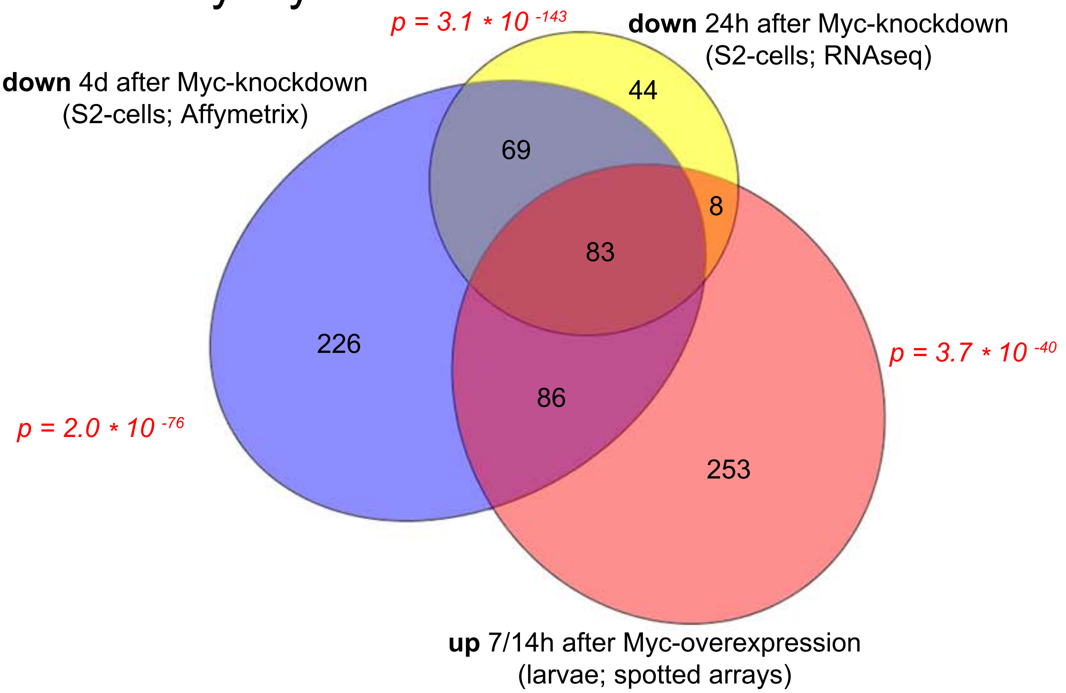

B: genes repressed by Myc

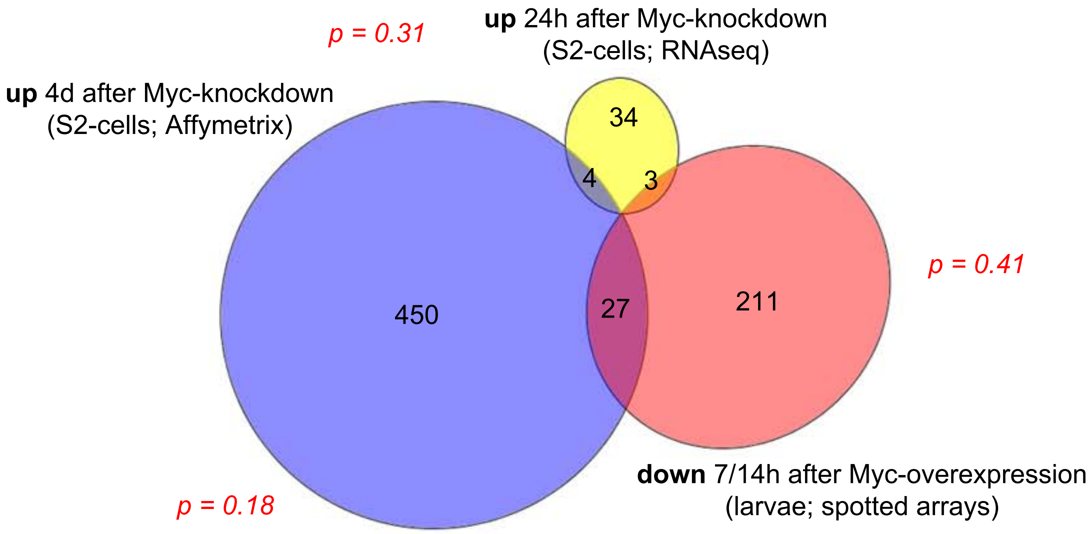

Sup. Figure 4

A

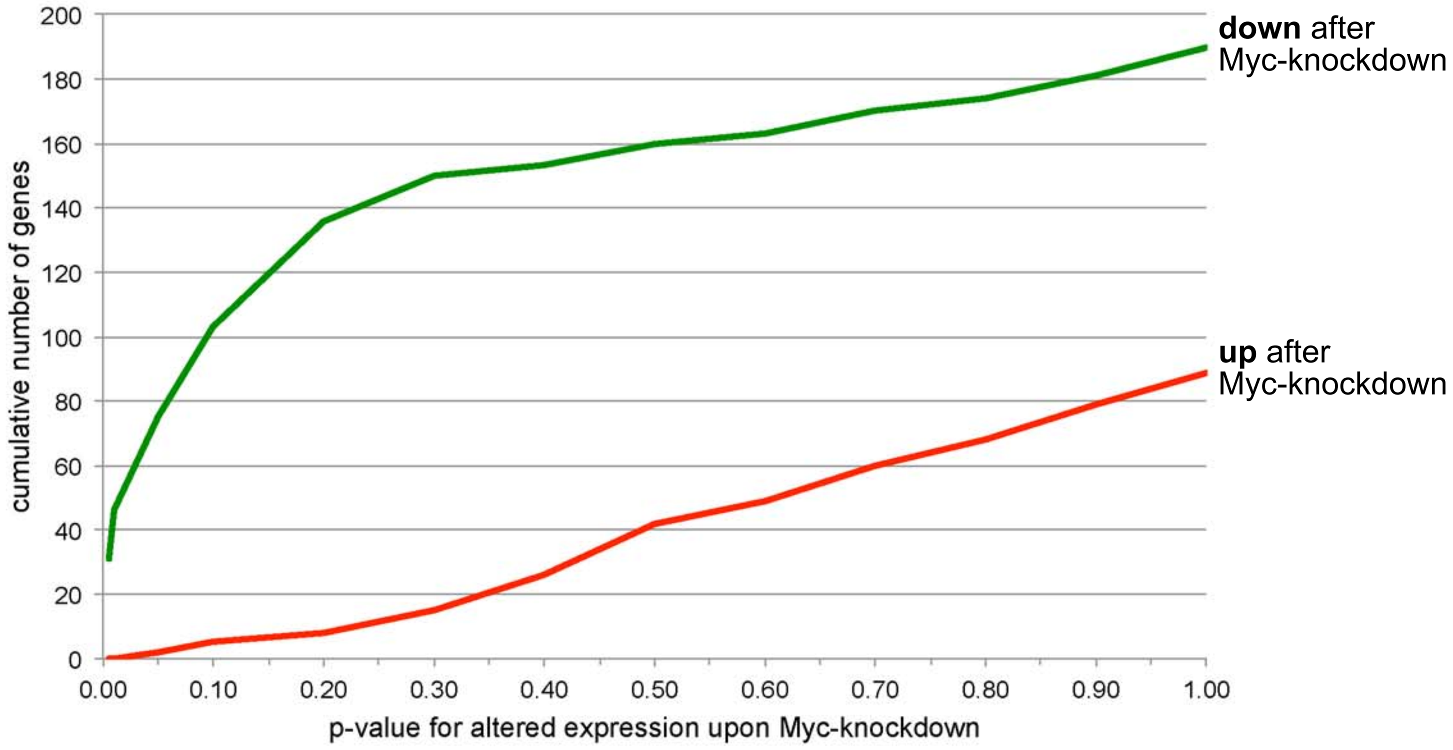

B

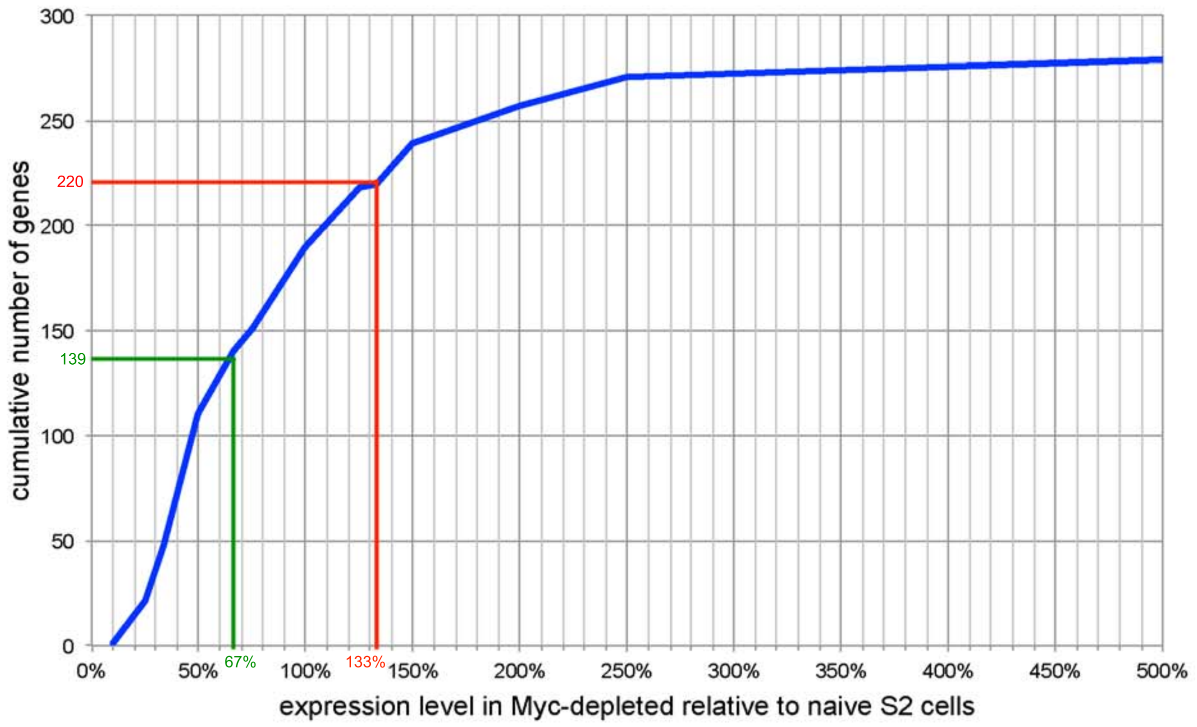

Sup. Figure 5

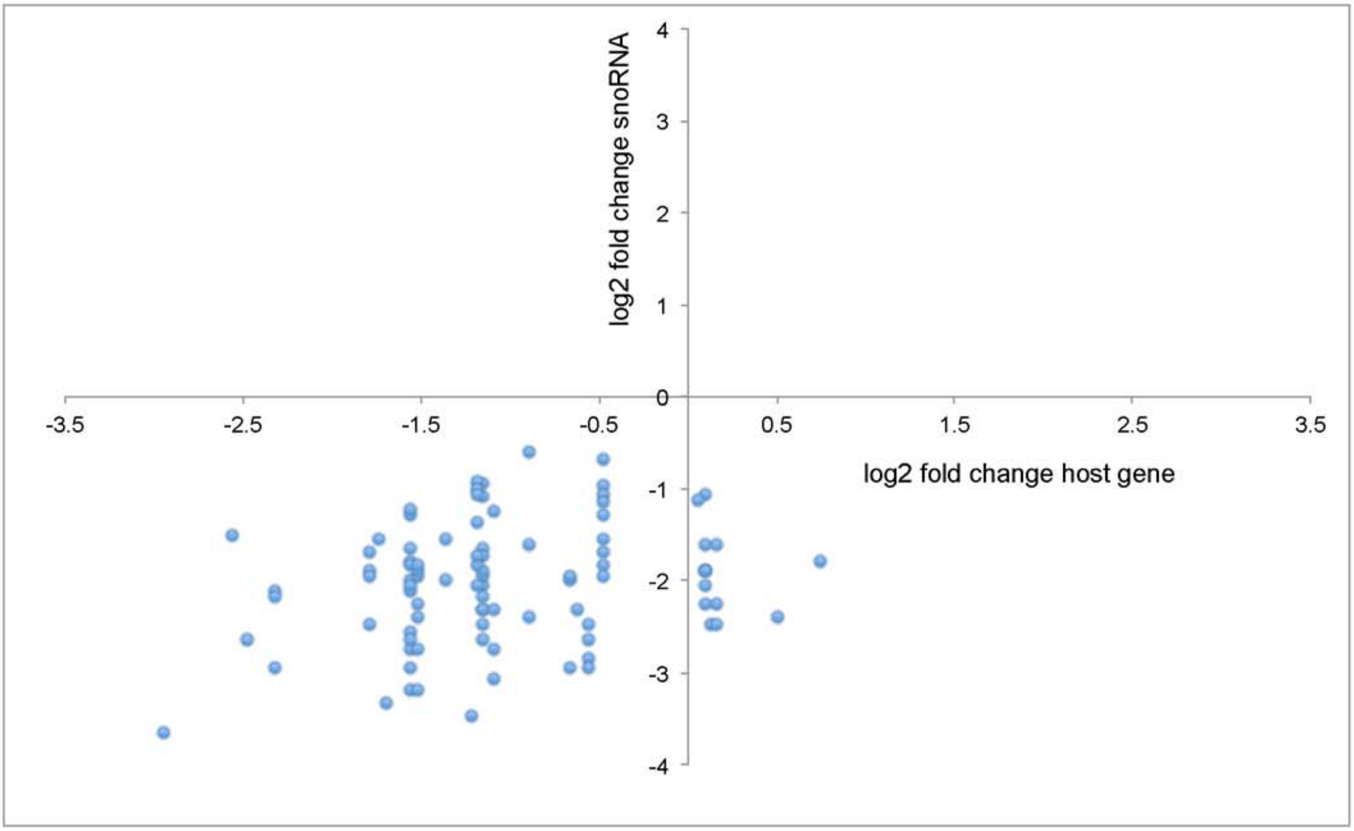

log2 fold change snoRNA
log2 fold change host gene
4
3
2
1
0
-1
-2
-3
-4
-3.5
-2.5
-1.5
-0.5
0.5
1.5
2.5
3.5

Sup. Figure 6

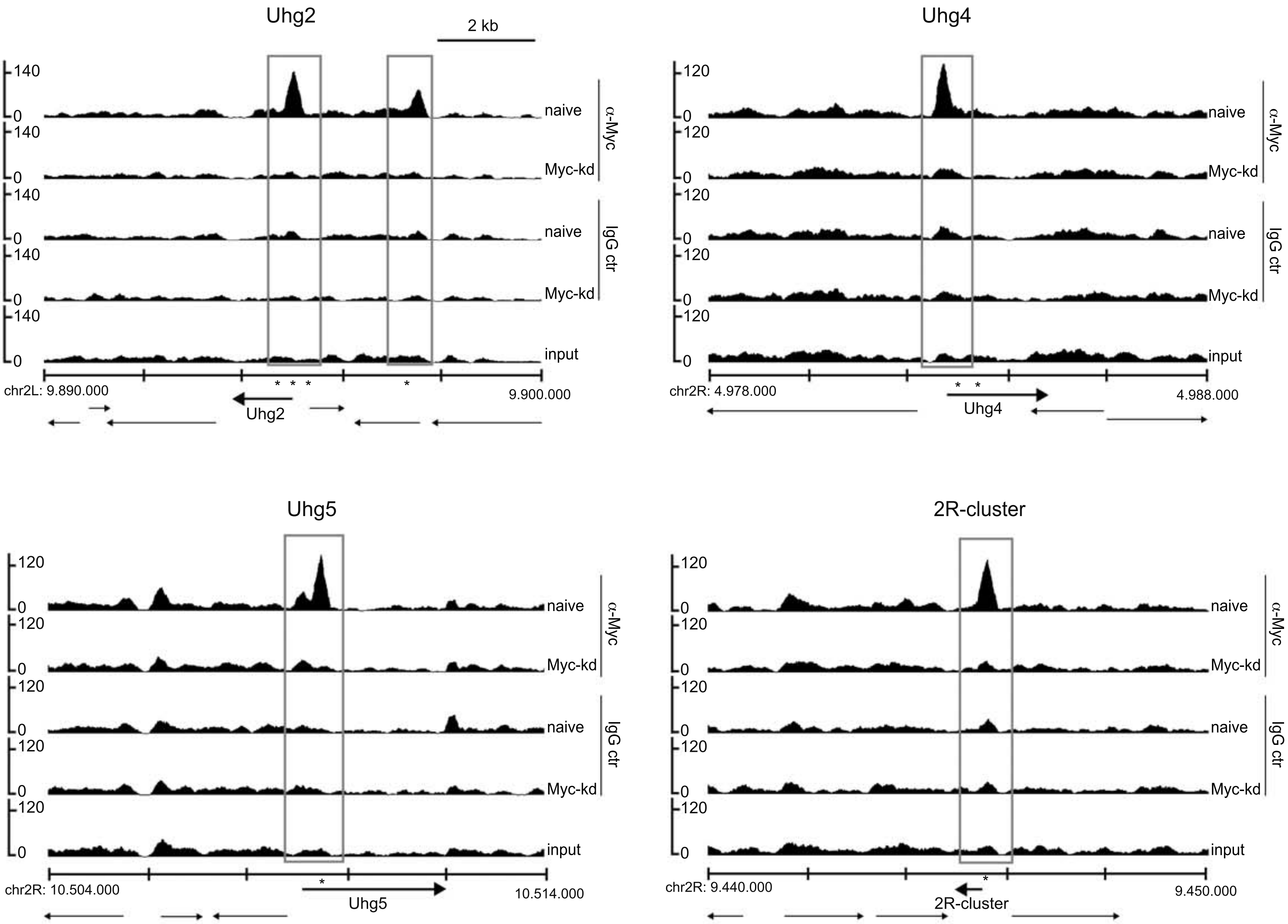

Uhg2
2 kb
140
0
naive
Myc-kd
naive
Myc-kd
input
α-Myc
IgG ctr
chr2L: 9.890.000
9.900.000
Uhg2
Uhg4
120
chr2R: 4.978.000
4.988.000
Uhg4
Uhg5
chr2R: 10.504.000
10.514.000
Uhg5
2R-cluster
chr2R: 9.440.000
9.450.000
2R-cluster

Sup. Figure 7

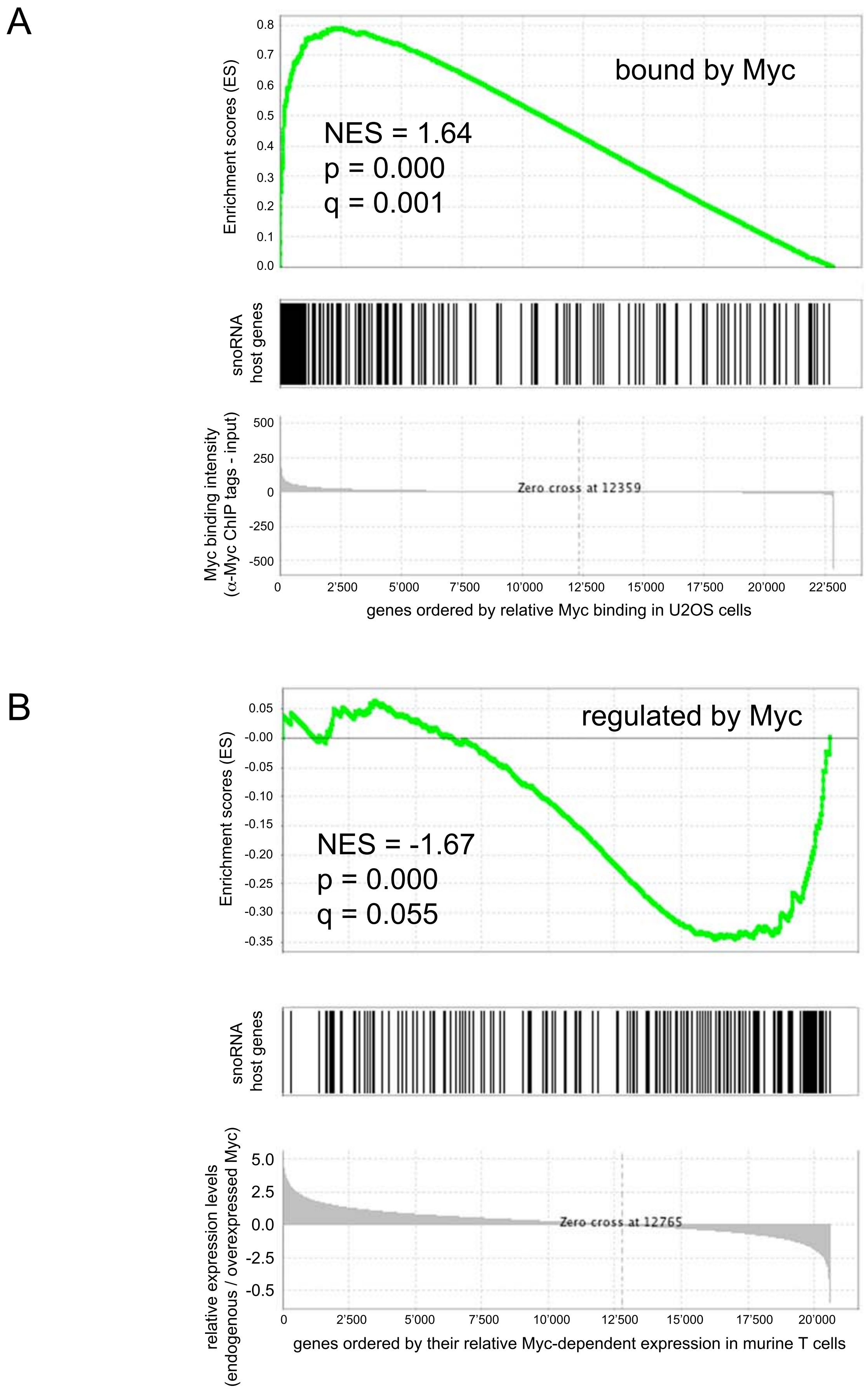
A
Enrichment scores (ES)
0.8
0.7
0.6
0.5
0.4
0.3
0.2
0.1
0.0
bound by Myc
NES = 1.64
p = 0.000
q = 0.001
snoRNA host genes
Myc binding intensity (α-Myc ChIP tags - input)
500
250
0
-250
-500
Zero cross at 12359
0
2'500
5'000
7'500
10'000
12'500
15'000
17'500
20'000
22'500
genes ordered by relative Myc binding in U2OS cells
B
Enrichment scores (ES)
0.05
-0.00
-0.05
-0.10
-0.15
-0.20
-0.25
-0.30
-0.35
regulated by Myc
NES = -1.67
p = 0.000
q = 0.055
snoRNA host genes
relative expression levels (endogenous / overexpressed Myc)
5.0
2.5
0.0
-2.5
-0.5
Zero cross at 12765
0
2'500
5'000
7'500
10'000
12'500
15'000
17'500
20'000
genes ordered by their relative Myc-dependent expression in murine T cells

Sup. Figure 8

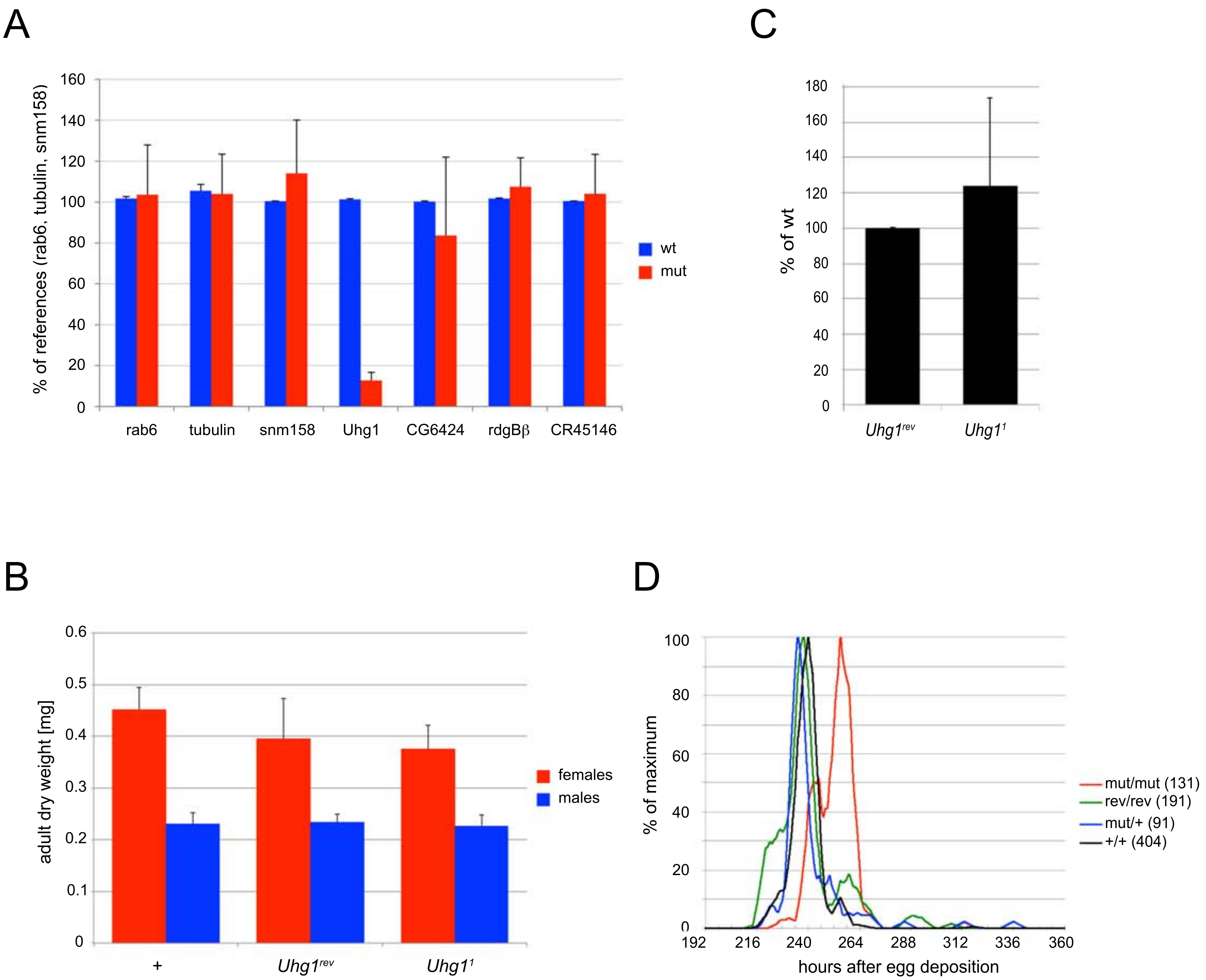
A
% of references (rab6, tubulin, snm158)
160
140
120
100
80
60
40
20
0
rab6
tubulin
snm158
Uhg1
CG6424
rdgBβ
CR45146
wt
mut
C
% of wt
180
160
140
120
100
80
60
40
20
0
Uhg1rev
Uhg11
B
adult dry weight [mg]
0.6
0.5
0.4
0.3
0.2
0.1
0
+
Uhg1rev
Uhg11
females
males
D
% of maximum
100
80
60
40
20
0
192
216
240
264
288
312
336
360
hours after egg deposition
mut/mut (131)
rev/rev (191)
mut/+ (91)
+/+ (404)
